# Anterior default mode network and posterior insular connectivity is predictive of depressive symptom reduction following serial ketamine infusion

**DOI:** 10.1017/S0033291722001313

**Published:** 2022-09

**Authors:** Benjamin S. C. Wade, Joana Loureiro, Ashish Sahib, Antoni Kubicki, Shantanu H. Joshi, Gerhard Hellemann, Randall T. Espinoza, Roger P. Woods, Eliza Congdon, Katherine L. Narr

**Affiliations:** 1 Ahmanson-Lovelace Brain Mapping Center, Department of Neurology, University of California, Los Angeles, CA, USA; 2 Department of Psychiatry and Biobehavioral Sciences, Semel Institute, UCLA, Los Angeles, USA

**Keywords:** Default mode network, functional connectivity, machine learning, major depressive disorder, serial ketamine infusion

## Abstract

**Background:**

Ketamine is a rapidly-acting antidepressant treatment with robust response rates. Previous studies have reported that serial ketamine therapy modulates resting state functional connectivity in several large-scale networks, though it remains unknown whether variations in brain structure, function, and connectivity impact subsequent treatment success. We used a data-driven approach to determine whether pretreatment multimodal neuroimaging measures predict changes along symptom dimensions of depression following serial ketamine infusion.

**Methods:**

Patients with depression (*n* = 60) received structural, resting state functional, and diffusion MRI scans before treatment. Depressive symptoms were assessed using the 17-item Hamilton Depression Rating Scale (HDRS-17), the Inventory of Depressive Symptomatology (IDS-C), and the Rumination Response Scale (RRS) before and 24 h after patients received four (0.5 mg/kg) infusions of racemic ketamine over 2 weeks. Nineteen unaffected controls were assessed at similar timepoints. Random forest regression models predicted symptom changes using pretreatment multimodal neuroimaging and demographic measures.

**Results:**

Two HDRS-17 subscales, the HDRS-6 and core mood and anhedonia (CMA) symptoms, and the RRS: reflection (RRSR) scale were predicted significantly with 19, 27, and 1% variance explained, respectively. Increased right medial prefrontal cortex/anterior cingulate and posterior insula (PoI) and lower kurtosis of the superior longitudinal fasciculus predicted reduced HDRS-6 and CMA symptoms following treatment. RRSR change was predicted by global connectivity of the left posterior cingulate, left insula, and right superior parietal lobule.

**Conclusions:**

Our findings support that connectivity of the anterior default mode network and PoI may serve as potential biomarkers of antidepressant outcomes for core depressive symptoms.

ClinicalTrials.gov: Biomarkers of Fast Acting Therapies in Major Depression, https://clinicaltrials.gov/ct2/show/NCT02165449, NCT02165449

## Introduction

Depression has a lifetime prevalence of over 20% (Hasin et al., 2018) making it a leading cause of disability. Compounding this, remission rates following common initial pharmaceutical interventions are about 30% with increasingly lower rates as patients fail to recover following subsequent interventions (Gaynes et al., 2009). An added bottleneck is the potential lag time between treatment initiation and the onset of antidepressant effect for most pharmaceutical and behavioral interventions. These circumstances stress the importance of identifying treatment-response biomarkers to serve as potential targets and understand mechanisms to guide more effective interventions, especially for rapidly-acting antidepressant treatments.

Ketamine is an N-methyl-d-aspartate receptor antagonist shown to induce robust and rapidly-acting antidepressant effects when administered in subanesthetic doses (Berman et al., 2000; Zarate et al., 2006). Ketamine's antidepressant effects occur within hours and cumulatively over days (Aan Het Rot, Zarate, Charney, & Mathew, 2012). Though the antidepressant effects of a single intravenous ketamine treatment are short-lived, repeated infusions performed roughly 2–3 times weekly can prolong these effects to weeks and longer (Murrough et al., 2013; Shiroma et al., 2014; Singh et al., 2016). Ketamine is a relatively novel antidepressant treatment, thus its effects on neural structure or function remain under active investigation. Relatively few studies have specifically investigated treatment-response biomarkers. Despite different methodologies employed across studies, a recent qualitative review (McMillan & Muthukumaraswamy, 2020) highlights that ketamine infusion generally preserves or enhances cortico-subcortical connectivity patterns captured by resting state functional connectivity (RSFC) (Dandash et al., 2015; Grimm et al., 2015; Höflich et al., 2015), while corticocortical connectivity is widely disrupted (Bonhomme et al., 2016; Joules et al., 2015; Schroeder et al., 2016). A recent review by Alario and Niciu surveyed genetic, RSFC, neurophysiological predictors of response to ketamine. The authors reported that ketamine generally normalized disrupted functional connectivity in patients with major depressive disorder, though study-specific results have varied and have largely failed to be replicated. Despite this, connectivity of the insula, anterior cingulate, and left amygdala was widely reported in relation to response to ketamine (Alario & Niciu, 2021). A recent study by our group using Arterial Spin Labelling (ASL) suggests perfusion of the bilateral hippocampus and insula is reduced with serial infusions (Sahib et al., 2020a). A related study probing treatment-related changes in RSFC reported that ketamine normalized aberrant somatomotor and default mode network (DMN) connectivity. Serial infusions also reportedly reduce connectivity between the cerebellum and salience network (SN) (Sahib et al., 2020c). Using a Go/No-Go task, we identified decreased functional activity in regions involved in inhibitory tasks including the DLPFC and inferior frontal cortex following serial ketamine treatments. To date, studies investigating predictors of clinical outcomes following ketamine infusion have been largely correlative rather than predictive. However, a pilot study by our group in an unrelated cohort found that symptom improvement 24 h following a single infusion of ketamine was related to pretreatment white matter integrity of the cingulum and forceps minor (Vasavada et al., 2016). A study by Abdallah et al. reported more robust antidepressant effects of ketamine in patients with smaller pretreatment left hippocampal volumes 24 h following treatment (Abdallah et al., 2015). In a task fMRI study, Murrough et al. reported that increased pretreatment connectivity of the right caudate while subjects were viewing positive emotional faces was associated with more reduced depressive symptoms following a single dose of intravenous ketamine (Murrough et al., 2015). Another study reported reduced connectivity between the lateral prefrontal cortex and subgenual anterior cingulate cortex associated with favorable antidepressant response following a single dose of ketamine (Gärtner et al., 2019). Another study identified that baseline cerebral blood flow of the fusiform and visual cortex was related to single and serial infusion response likelihood, respectively (Sahib et al., 2020a).

Although its multifaceted etiology is poorly understood, depression is widely characterized as a brain network disorder involving disrupted RSFC of large-scale resting state networks (RSNs) including the DMN, SN, and central executive network (CEN) (Kaiser, Andrews-Hanna, Wager, & Pizzagalli, 2015; Menon, 2011; Sheline, Price, Yan, & Mintun, 2010). Aberrant activity of these RSNs may be mediated in part by structural abnormalities of specific subregions or microstructural alterations of connective white matter. Converging evidence suggests that patients with depression exhibit increased connectivity within the anterior DMN, increased connectivity between the SN and anterior DMN, altered connectivity between anterior and posterior DMN, and decreased connectivity between the CEN and posterior DMN (Mulders, van Eijndhoven, Schene, Beckmann, & Tendolkar, 2015).

Structural neuroimaging has highlighted reduced hippocampal volumes depressed patients (Schmaal et al., 2016). The hippocampus is broadly involved in learning and memory as well as emotional regulation processes relevant to depression (Gulyaeva, 2015). Due to its high concentration of glucocorticoid receptors, it is also highly prone to stress-related atrophy mediated by the release of circulating glucocorticoids by the hypothalamic–pituitary–adrenal axis (Smith & Vale, 2006). Also widely implicated in depression pathophysiology are structural abnormalities of the anterior cingulate, prefrontal cortex, and amygdala (Drevets, Savitz, & Trimble, 2008; Lorenzetti, Allen, Fornito, & Yücel, 2009; Schmaal et al., 2016). Numerous microstructural abnormalities of white matter tracts have also been identified in depression including the superior longitudinal fasciculus (SLF), uncinate fasciculus, corona radiata, and cingulum (Kieseppä et al., 2010; Korgaonkar et al., 2011; Murphy & Frodl, 2011; Wang, Leonards, Sterzer, & Ebinger, 2014). Whether these observations of structural and functional imaging abnormalities commonly presented in patients with depression predict antidepressant response to ketamine remains unknown.

Univariate, correlative studies have done much to advance our understanding of treatment-response biomarkers. However, robust biomarkers may lie within a multivariate space. Further, putative biomarkers should be validated using hold-out data. Thus, machine learning models trained and tested using rigorous cross-validation offer a means by which to directly evaluate the predictive validity of potential multivariate biomarkers. The identification of biomarkers predictive of antidepressant outcomes following serial ketamine infusion (SKI) may be of substantial benefit for advancing the development of personalized treatment strategies for ketamine and potentially also for other fast-acting therapies. Here, we evaluated 60 patients with major depression undergoing a series of four ketamine infusions and 19 unaffected and untreated controls. We constructed purely data-driven machine learning models to predict individual reductions in depressive symptoms following treatment using pretreatment multimodal RSFC, structural neuroimaging, and demographic data. We hypothesized that pretreatment RSFC of the DMN and SN as well as measures of key limbic structures such as the hippocampus would be most informative of clinical outcomes following SKI.

## Methods and materials

### Participants

Sixty patients experiencing a major depressive episode diagnosed by the Structured Clinical Interview for DSM-V were assessed between January 2017 and April 2020. Nineteen unaffected controls were included to determine whether ketamine significantly reduced symptoms compared to an untreated cohort. Table 1 details patient characteristics. Notably, participants overlap with previous studies (Loureiro et al., 2020; Sahib et al., 2020b, 2020c; Vasavada et al., 2020). Patient inclusion criteria included failure to respond adequately to at least two prior antidepressant medications, age between 20 and 64 years, DSM-5 diagnosis of major depression, a current episode of depression lasting for at least 6 months, 17-item Hamilton Depression Rating Scale (HDRS) scores ⩾17, and stable antidepressant or mood stabilizer use (i.e. no treatment changes) for at least 6 weeks. Exclusionary criteria included rapid-cycling bipolar disorder, psychotic reactions to medication, intellectual disability or developmental disorders, comorbid substance abuse in the past 3 months, diagnosis of schizophrenia/schizoaffective disorder, Alzheimer's disease, or receipt of neuromodulation or ketamine treatment in the past 6 months. All patients underwent MRI scanning and clinical assessments within one week of their first treatment and again 24 h following the end treatment (if the last treatment occurred on a Friday, assessments occurred 72 h post treatment on a Monday). Depressive and ruminative symptoms were assessed before and after treatment using the HDRS-17, Inventory of Depressive Symptomatology (IDS-C), 16-item Quick Inventory of Depressive Symptomatology Self Report (QIDS-SR), and the 10-item Rumination Response Scale (RRS) (Treynor, Gonzalez, & Nolen-Hoeksema, 2003). Control participant symptoms were assessed twice approximately 2–3 weeks apart using the same scales (except the RRS). Inclusion criteria for non-depressed control participants were age between 20 and 64 years, no history of depressive disorder or bipolar disorder that is current, recurrent, or with a single episode that lasted longer than one year, no use of antidepressants or mood stabilizers within the past 6 months, ability to read and understand English, and an ability to provide informed consent. Exclusionary criteria for controls included substance abuse in the past 3 months, diagnosis of schizophrenia/schizoaffective disorder, prior use of antidepressant mood stabilizers, developmental disorders, or a diagnosis of dementia. All participants received measurement of vital signs, a blood draw to determine metabolic, kidney, and liver function, EKG, and provided a urine sample for drug and pregnancy (women only) screening. Drug and pregnancy screens were required to be negative, and all lab results were reviewed by the study physician to ensure there were no contraindications to participating in the study. All participants provided written informed consent following procedures approved by the UCLA Institutional Review Board (IRB).

**Table 1.** Demographic and clinical outline

	Patients	Controls	*p* (*T* or χ^2^)
*N*	60	19	
Age, mean (s.d.)	40.1 (11.1)	28.2 (6.8)	<0.0001
Sex, male/female	30/30	8/11	0.73
Lifetime illness duration, years, mean (s.d.)	24.8 (15.9)	N/A	–
Current illness duration, years, mean (s.d.)^a^	4.0 (5.9)	N/A	–
Number of lifetime depressive episodes, mean (s.d.)^b^	6.8 (14.4)	N/A	–
Bipolar disorder, yes/no	3/57	N/A	–
Clinical rating scales			
HDRS-17 baseline, mean (s.d.)	18.9 (4.6)	2.6 (5.1)	<0.0001
HDRS-17 change, mean (s.d.)	−10.6 (6.3)	−0.2 (2.1)	<0.0001
QIDS-SR baseline, mean (s.d.)	16.1 (4.8)	4.2 (6.2)	<0.0001
QIDS-SR change, mean (s.d.)	−9.5 (5.1)	−0.5 (1.3)	<0.0001
IDS-C baseline, mean (s.d.)	30.5 (7.4)	5.1 (9.4)	<0.0001
IDS-C change, mean (s.d.)	−17.6 (10.7)	−0.5 (2.7)	<0.0001
HDRS-17 responder/non-responder, *N*	37/23	N/A	–
HDRS-17 remitter/non-remitter, *N*	30/30	N/A	–
Medication history			
Current SSRI use, yes/no	18/42	N/A	–
Current SNRI use, yes/no	18/42	N/A	–
Current MAOI use, yes/no	2/58	N/A	–
Current lithium use, yes/no	1/59	N/A	–
Current benzodiazepine use, yes/no	16/44	N/A	–
Current antipsychotic use, yes/no	13/47	N/A	–
Current use of any medication, yes/no	48/12	N/A	–
Education level, *N*^c^			
High school or equivalent	3	0	–
Some college	23	3	–
Bachelor's	23	9	–
Masters'	14	3	–
Professional or doctoral	6	3	–
Abbreviations: HDRS: Hamilton Depression Rating Scale; QIDS: Quick Inventory of Depressive Symptomatology; IDS: Inventory of Depressive Symptomatology; SSRI: Selective Serotonin Reuptake Inhibitor; SNRI: Serotonin and Norepinephrine Reuptake Inhibitor; MAOI: Monoamine Oxidase Inhibitor			

a Three participants reported uncertain illness durations and were excluded from this calculation.

b Thirty-five participants reported more episodes than they could count and were excluded from this calculation.

c One patient and one control had incomplete data on education level.

### SKI treatment

Patients received a total of four ketamine infusions spaced 2–3 days apart. Ketamine was administered in subanesthetic doses (0.5 mg/kg) diluted in 60 cc of saline and delivered intravenously via a pump over a 40 min session at the UCLA Clinical and Translational Center Research (CTRC) or Resnick Neuropsychiatric Hospital. Vital signs including blood pressure, pulse oximetry, and respiratory rates were monitored during ketamine administration. Patients receiving ketamine were allowed to continue preapproved monoaminergic antidepressant medications, i.e. serotonin, norepinephrine, and dopamine reuptake inhibitors, and tricyclics; however, benzodiazepines were discontinued 24 h prior to all scanning and treatment sessions.

### Image acquisition and preprocessing

Images were acquired using a Siemens 3 T Prisma MRI system at UCLA's Brain Mapping Center with a 32-channel phased array head coil. Acquisition sequences were identical to the Human Connectome Project Lifespan studies for Aging and Development (https://www.humanconnectome.org). Detailed acquisition parameters, processing steps, and extraction of multimodal imaging measures are outlined in online Supplementary methods.

### Predictive features

Demographic (age and sex) and pretreatment multimodal imaging features were included as predictors.

Multimodal imaging data were visually inspected and preprocessed using HCP minimal pipelines (Glasser et al., 2013; Smith et al., 2013) with the BIDS-App (Gorgolewski et al., 2017). Functional imaging artifacts were removed using a modified spatial independent components analysis method (Griffanti et al., 2014) and FSL's FIX (https://fsl.fmrib.ox.ac.uk/fsl/fslwiki/FIX). MSMall registration aligned cortical regions using measures of cortical folding, thickness, myelination, and resting-state connectivity information (Glasser et al., 2016; Robinson et al., 2018, 2014). Resting-state time series data were represented on the cortical surface and the time series was averaged across AP and PA acquisitions. Structural imaging features included regional estimates of cortical thickness in 68 regions based on the Desikan-Killiany atlas (Desikan et al., 2006), 24 subcortical volumes, diffusion measures [fractional anisotropy (FA), radial (RD), axial (AD), mean diffusivity (MD), and diffusion kurtosis (DK)] of 48 white matter tracts. NiLearn scripts were used to compute functional global connectivity measures of 360 cortical and 21 subcortical regions. Subjectwise correlation matrices were thresholded at *r*≥|0.3| to create a binary adjacency matrix for each subject. The node degree of each regional parcellation was computed as the number of other regions with which a given region was correlated above the threshold of *r*≥|0.3|, providing a proxy of regional global connectivity. A tabulation of regional predictors is included in online Supplementary Table S1.

### Clinical outcome measures

Depression is a symptomatically heterogeneous disorder. The diversity of potential symptom profiles motivates the evaluation of treatment outcomes along multiple symptom dimensions. Thus, we constructed models to predict change along multiple scales: the 17- and 6-item HDRS (Bech et al., 1981), the IDS-C, the QIDS-SR, the brooding and rumination dimensions of the RRS (RRSB and RRSR), and three symptom dimensions of the 17-item HDRS identified previously (Wade et al., 2020, 2021): core mood and anhedonia (CMA), somatic disturbances (SOD), and insomnia. Subscales of the HDRS-17 used here are outlined in Table 2.

**Table 2.** Subscales of the Hamilton Depression Rating Scale

HDRS-17 items	HDRS-6	Core mood and anhedonia	Somatic disturbances	Insomnia
Depressed mood	X	X		
Feelings of guilt	X		X	
Suicide				
Insomnia, early				X
Insomnia, middle				X
Insomnia, late				X
Work and activities	X	X		
Psychomotor retardation	X	X		
Agitation			X	
Anxiety, psychic	X		X	
Anxiety, somatic			X	
Somatic symptoms, GI			X	
Somatic symptoms, general	X		X	
Genital symptoms			X	
Hypochondriasis			X	
Weight loss		X		
Insight				

### Predictive modeling

We trained separate random forest regression (RFR) models, each with 5000 underlying regression trees, to predict change along each symptom set using pretreatment predictors outlined above. Models were trained and validated using 10-repeated 10-fold cross-validation. The primary measure of model performance was the sums of squares formulation of the *R*^2^; i.e. 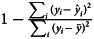, where 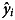 and 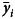
are the predicted outcome and the *i*-th subject and the average outcome across all subjects in the testing folds, respectively (Poldrack, Huckins, & Varoquaux, 2020). We additionally report the normalized RMSE (NRMSE) value to facilitate comparisons across scales with different ranges:  
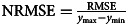
 where *y*_max_ and *y*_min_ are the maximum and minimum outcome values, respectively, in the test observations. The significance of each model's performance was assessed using permutation tests (100 resamples). We adjusted for multiple comparisons across the set of symptom sets using the standard FDR approach.

## Results

### Cohort characteristics

Sixty patients (age = 40.1±11.1 years, *n* = 30 males) were included. Patient age was associated with several outcome measures: the HDRS-6, QIDS-SR, IDS-C, and the SoD dimension (all *p* < 0.05) with older patients showing a greater reduction in symptoms, on average, though sex was not significantly associated with outcomes. Using the HDRS-17 criterion of 50% symptom reduction, 37 (61%) patients experienced clinical response following SKI and 30 (50%) patients achieved remission (defined as a post-treatment HDRS-17 total score ≤7). We used unpaired, two-sample *t* tests to compare the degree of symptom changes between patients and controls. As expected, symptoms captured by the HDRS-17, IDS-C, and QIDS-SR were significantly more reduced among patients than controls (all *p* < 0.0001).

### Model performance

**Fig. 1.**
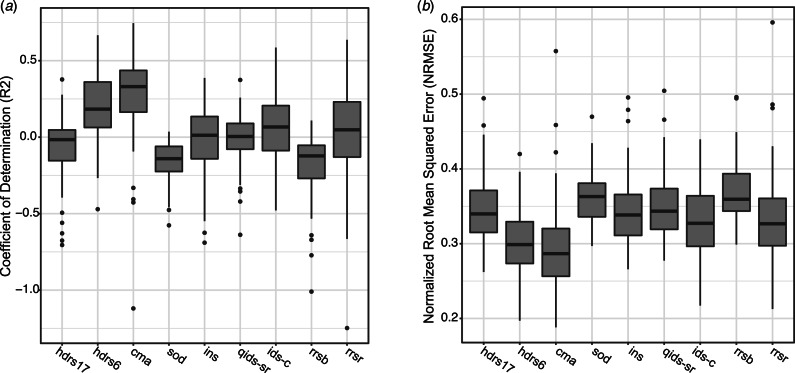
Boxplots of cross-validated model performance. (*a*) Shows distributions of the sums-of-squares formulation of the coefficient of determination (*R*^2^) in test data across 100 iterations of cross-validation while (*b*) shows the normalized root mean squared error of predictions across cross-validations.

In patients, HDRS-6 and CMA change were predicted most accurately with *R*^2^ scores of 0.19 (*q* < 0.05) and 0.27 (*q* < 0.05), respectively. The RRSR scale was predicted with a modest but significant *R*^2^ score of 0.01 (*q* < 0.05). No other symptom sets were predicted significantly above chance after adjustment for multiple comparisons. Model performance measures (*R*^2^ and NRMSE) are outlined in Fig. 1 and Table 3, while plots of predicted *v.* actual symptom changes are illustrated in online Supplementary Fig. S1.

**Table 3.** Model performance

Outcome	*R*^2^	NRMSE	Adjusted *p* value (*R*^2^)
HDRS-17	−0.06	0.34	0.20
HDRS-6	0.19	0.30	0.02
CMA	0.27	0.29	0.02
SOD	−0.15	0.36	0.72
INS	−0.01	0.34	0.29
QIDS-SR	−0.01	0.35	0.07
IDS-C-M	0.04	0.33	0.06
RRSB	−0.17	0.36	0.72
RRSR	0.01	0.33	0.02

### Features informative of outcomes

**Fig. 2.**
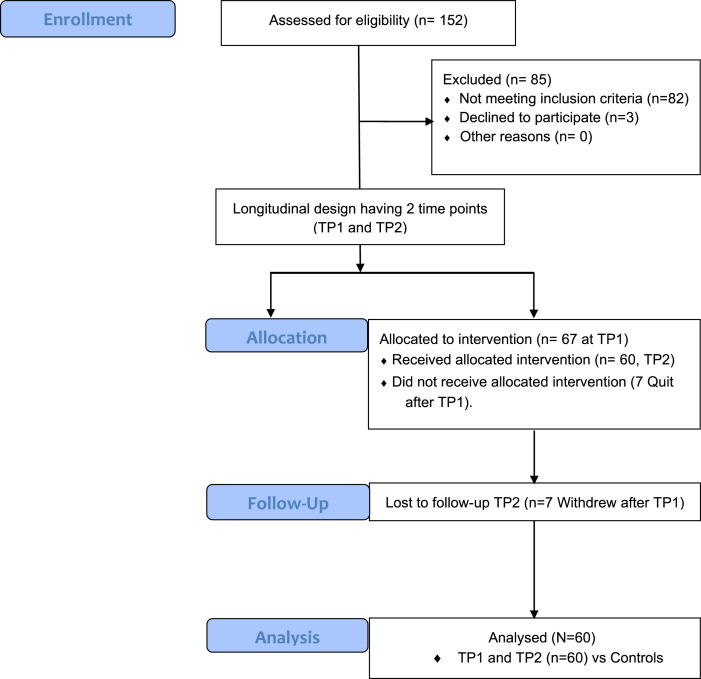
Partial dependence plots showing the expected change in symptoms (*y*-axis) for observed values of informative imaging predictors (*x*-axis) while averaging other predictors. Associations between pretreatment values of the most informative predictors and expected symptom changes are shown for (*a*) HDRS-6 (top row) and core mood and anhedonia (middle row) symptoms. The bottom row illustrates the location of the right posterior insula area 2 (PoI2), right anterior cingulate/medial prefrontal cortex (BA 10r), and right superior longitudinal fasciculus (SLF). Figure (*b*) illustrates the same for the rumination response scale: reflection associations with the posterior cingulate (v23ab subdivision), superior parietal cortex (7PL subdivision), and the granular insular cortex.

The most informative features in the prediction of HDRS-6 and CMA changes were the global connectivity of the right posterior insular area 2 (PoI2) and the right Brodmann area (BA) 10r comprising the anterior cingulate cortex (ACC) and medial prefrontal cortex (mPFC), and the DK of the right SLF. In Fig. 2 we show partial dependence plots that illustrate the expected symptom changes for important predictors while holding all other predictors in the model at observed constants. These show that increased global connectivity of areas PoI2 and 10r predicted greater reduction in HDRS-6 and CMA symptoms while increased DK of the SLF predicted less reduction of HDRS-6 and CMA symptoms.

Change in the RRSR scale was predicted by the pretreatment global connectivity of the right BA 7PL (posterolateral BA 7; superior parietal cortex), the left insular gyrus, and left v23ab subdivision of the posterior cingulate cortex (PCC) and right putamen volume. Higher global connectivity of right BA 7PL, left insular gyrus, and left area v23ab along with higher right putamen volume predicted poorer reduction of ruminative symptoms.

### Evaluation of confounding variables

We evaluated whether predictive imaging measures outlined above (PoI2, BA 10r, SLF DK, BA 7PL, left insular gyrus, and left v23ab) and significantly predicted outcomes were associated with potential confounding clinical and demographic variables. Measures of global connectivity are based on counts, thus we evaluated associations with potential confounding variables using Poisson regression. The diffusivity of the SLF and thickness of the left insular gyrus were normally distributed (following a Shapiro–Wilk test; *p* > 0.05) and continuous measures; thus, associations with confounds were evaluated using standard linear regression models. Global connectivity of the right PoI2 was associated with age, sex, education, duration of current episode, and current SSRI, SNRI, and benzodiazepine use (all *p* < 0.05). Global connectivity of right BA 10r was associated with age, sex, duration of current episode, and current SNRI use (all *p* < 0.05). Global connectivity of right BA 7PL was associated with sex, education, current episode duration, and current SSRI use (all *p* < 0.05). Left area v23ab was associated with current episode duration, and current use of antipsychotics and SNRIs (all *p* < 0.05). The kurtosis of the right SLF was not associated with the evaluated confounding variables. The left insula was associated with patient age and SSRI use (all *p* < 0.05). We reran the original RFR models with all of the original predictors with the addition of medication use history, depressive episode duration, and education level. The *t* tests revealed no significant differences between original and updated model performance upon inclusion of additional confounding variables (all *p* > 0.9). Further, no potential confounding clinical or demographic variables were among important predictors in the updated models. Thus, despite their associations with important imaging predictors, these potentially confounding variables did not improve prediction of outcomes for any dimension.

## Discussion

We explored whether changes in depressive symptoms following SKI were predictable using multivariate patterns of pretreatment RSFC and demographic variables. Because depression is a symptomatically heterogeneous disorder, we evaluated whether symptomatic changes were predictable across multiple depression scales and HDRS subscales. We observed a wide spread of performances across outcomes suggesting that changes along certain symptom clusters are more robustly predictable than others. In particular, symptom changes along the HDRS-6 and CMA symptom clusters were predicted most accurately. Notably, these are two subscales of the HDRS-17 with three overlapping items: depressed mood, work and activities, and psychomotor retardation. Similarly, change along these two scales was informed most by the pretreatment global connectivity of the right PoI2 and BA 10r spanning the anterior cingulate and mPFC, and diffusivity of the SLF.

Right BA 10r encompasses portions of the ACC and mPFC; components of the anterior DMN. Previous depression studies have generally reported decreased connectivity of the anterior and posterior components of the DMN. For example, van Tol reported decreased connectivity of the dorsal mPFC with posterior portions of the DMN including the precuneus, angular gyrus, and middle temporal gyrus in depression relative to unaffected controls (van Tol et al., 2014). The same study reported elevated connectivity between the dorsal mPFC and components of the SN: the insula and superior frontal gyrus. Several studies have investigated connectivity of the anterior DMN using independent components analysis (ICA). Among these, the anterior DMN has been consistently reported to be hyperconnected in patients relative to controls (Greicius et al., 2007; Mulders et al., 2015; Zhu et al., 2012). In related work, Abdallah et al. identified no differences in regional global connectivity between subsequent responders and non-responders to single-infusion ketamine, however, ketamine responders showed increased change in connectivity of the right lateral PFC with treatment (Abdallah et al., 2017). Notably, a previous study showed that RSFC between the pregenual ACC and right lateral PFC was significantly associated with the extent of symptom reduction 24 h after single ketamine infusion therapy (racemic and S-ketamine) in major depression in line with our findings (Gärtner et al., 2019). A double-blind, placebo-controlled, crossover study reported that connectivity between the insula and DMN was normalized compared to controls 2 days following a single ketamine infusion (Evans et al., 2018), further implicating regions identified here. A combined EEG/fMRI study by Zacharias in healthy volunteers reported that ketamine reduced connectivity of the anterior DMN (Zacharias et al., 2020). Similarly, Li et al. reported that a single infusion of ketamine reduced connectivity between the PCC and the dorsomedial prefrontal cortex in 61 healthy participants (Li et al., 2020). Apart from ketamine, RSFC of anterior DMN components have been reported to be predictive of antidepressant response to first-line antidepressant medications (Kozel et al., 2011), cognitive behavioral therapy (Dunlop et al., 2017), transcranial magnetic stimulation (Fox, Buckner, White, Greicius, & Pascual-Leone, 2012; Salomons et al., 2014), and electroconvulsive therapy (van Waarde et al., 2015).

The right PoI2 is a subdivision of the posterior insula and frontal opercular complex and is a component of the ventral attention network, (VAN) DMN, and the externally directed CEN. The functional role of the posterior insula has largely been ascribed to sensorimotor tasks with affective significance (Craig, 2009; Kurth, Zilles, Fox, Laird, & Eickhoff, 2010). Hu et al. used amplitude of low-frequency fluctuation analysis investigate intrinsic neural oscillation abnormalities of the insula in adolescence with depression and reported decreased activity of the right posterior insula in youth with depression relative to unaffected controls (Hu et al., 2019). A follow-up seed-based analysis in the same cohort revealed reduced functional connectivity between the right posterior insula and several visual, somatomotor, and limbic regions (Hu et al., 2019). In a cohort of 19 patients with depression and 19 controls, Peng et al. reported on decreased functional connectivity between the right posterior insula and the posterior parietal cortex, part of the CEN, in patients relative to controls (Peng et al., 2018). Ketamine has also been shown to increase the global connectivity of the insula in responsive patients (Abdallah et al., 2017). An open-label magnetoencephalography study of treatment-related effects of ketamine reported a reduction in connectivity between an insulo-temporal ICA component and the amygdala (Nugent, Robinson, Coppola, & Zarate, 2016). In an overlapping sample, using positron emission tomography, the same group reported decreased metabolism of the insula following a single dose of ketamine (Carlson et al., 2013). These findings support that the insula may mediate specific symptom expressions in depression while our current findings support that disruptions in these same regions may be related to treatment response likelihood. Indeed, one study has shown RSFC between the pregenual ACC and insula predicts differential outcomes following treatment with medication and cognitive behavioral therapy in major depression (Dunlop et al., 2017).

The pretreatment DK of the SLF diffusion was predictive of both HDRS-6 and CMA changes. The SLF has widely been associated with depressive symptoms (de Diego-Adeliño et al., 2014; Jiang et al., 2017; Korgaonkar et al., 2011; Reppermund et al., 2014) and in several studies, FA of this tract was found inversely correlated with the severity of depressive symptoms (Lai & Wu, 2014) and illness duration (de Diego-Adeliño et al., 2014; Gu et al., 2013). A recent meta-analysis of microstructural abnormalities in medication-free patients with depression identified robust FA reductions in the SLF III (Jiang et al., 2017), which connects the supramarginal gyrus and ventral premotor and prefrontal cortices. The connectivity of this tract suggests that it plays a role in processing somatosensory information (Makris et al., 2005). As psychomotor retardation is a symptom captured both by the HDRS-6 and CMA symptom dimensions, this offers a plausible link between the SLF and its role as a potential biomarker of symptom reduction in these related dimensions. Increased DK reflects an increasingly complex microstructural environment, implying more obstacles to normal diffusion (Steven, Zhuo, & Melhem, 2013), such as increased cell density or complexity, while lower DK may suggest atrophied cellular structure. Here, we observed more reduced HDRS-6 and CMA symptoms among patients with lower DK in the right SLF that may suggest that depression-related microstructural abnormalities of the SLF are indicative of subsequent responsivity to SKI. Related to these findings, a pilot study on 13 patients with treatment-resistant depression who received a single infusion of ketamine reported that higher FA of the left SLF was associated with better symptom improvement 24 h post-infusion (Sydnor et al., 2020).

Ruminative symptoms are commonly described by a two-factor model with subcomponents of brooding and reflection. Brooding has been more consistently linked with maladaptive cognitive traits of depression (Lo, Ho, & Hollon, 2008) and suicide ideation (Surrence, Miranda, Marroquín, & Chan, 2009); however, reflective symptoms are related to depression severity (Fresco, Frankel, Mennin, Turk, & Heimberg, 2002; Surrence et al., 2009). Here, change in reflective (RRSR) symptoms was predicted significantly. In a sample of 10 patients with treatment-resistant depression, Vidal et al. reported that a single infusion of ketamine was effective at reducing ruminative symptoms (Vidal, Jermann, Aubry, Richard-Lepouriel, & Kosel, 2020). Here, we saw that change in RRSR symptoms was predicted by pretreatment global connectivity of left ventral area 23ab (in the PCC), the left insular granular complex, area 7PL of the right superior parietal cortex, and the volume of the right putamen. Increased RSFC and volume was associated with less reduction of RRSR symptoms. The PCC is a hub of the DMN and hyperconnectivity of this region has widely been associated with increased ruminative symptoms (Berman et al., 2011; Cooney, Joormann, Eugène, Dennis, & Gotlib, 2010). This may suggest that increased connectivity of the PCC along with components of the dorsal attention (superior parietal cortex) and somatomotor networks (insular granular complex) may reflect increased treatment resistance to ketamine.

Lastly, it was hypothesized that hippocampal volume or connectivity would be a predictor of response to SKI given that it has been associated with response to other treatments including electroconvulsive therapy (Wade et al., 2017) and pharmaceutical treatments (Colle et al., 2018). Further, the effects of depression on hippocampal structure have been widely reported (Sheline, Wang, Gado, Csernansky, & Vannier, 1996) making its structural or functional properties a plausible candidate biomarker of treatment response. Contrary to our expectations, however, hippocampal structure and function was not implicated as a predictor of response to SKI in this multivariate framework. It is possible that this may reflect distinct mechanisms of actions for SKI or a predominating association of more salient predictors in a multivariate framework that overshadow the contributions of the hippocampus.

## Limitations

There are several limitations to consider in interpreting the findings of this study. Statistical and machine learning models are prone to overfitting to training data when the number of possible predictors exceeds the number of samples used to train the data. However, we used a conservative 10-repeated 10-fold cross-validation approach to directly evaluate model performance in hold-out data. Further, this was not a randomized clinical trial and lacked an active placebo group. Additional considerations include that patients were allowed to continue on current and stable antidepressant medication treatments; however, these were not associated with key predictors or outcomes.

## Conclusions

Using a purely data-driven approach and multimodal MRI, our study supports that pretreatment global connectivity of the ACC, mPFC, and posterior insula as well as diffusivity of the SLF are potential biomarkers of antidepressant outcomes following SKI. Importantly, these regions form nodes or structural connections encompassing the DMN and SN/VAN, and have been widely implicated in the pathology of depression thus adding to their plausibility in this role. We evaluated biomarkers of response along a number of widely-used symptom scales, sub-scales, and less commonly-used sub-scales identified by previous studies. We found that core symptoms of depression captured by the HDRS-6 and an overlapping dimension of CMA were predicted most accurately. Future work will investigate biomarkers of durable, long-term response to SKI. This work may advance the ultimate goal of using imaging or other physiological markers to personalize treatments to improve and speed recovery in individual patients.

## Supplementary material

For supplementary material accompanying this paper visit https://doi.org/10.1017/S0033291722001313. click here to view supplementary material
