# Supplementary material for: Anterior default mode network and posterior insular connectivity is predictive of depressive symptom reduction following serial ketamine infusion
Source: Psychol Med. 2022 May 17;52(12):2376–86. doi: 10.1017/S0033291722001313 (PMC9527672; doi:10.1017/S0033291722001313)
Supplement: Supplementary file 1 [file S0033291722001313sup.zip › S0033291722001313sup003.pdf]

Psychological Medicine

Anterior Default Mode Network and Posterior Insular Connectivity is Predictive of Depressive Symptom Reduction Following Serial Ketamine Infusion

--Manuscript Draft--

**Manuscript Number:**	PSM-D-21-01672R1
**Full Title:**	Anterior Default Mode Network and Posterior Insular Connectivity is Predictive of Depressive Symptom Reduction Following Serial Ketamine Infusion
**Article Type:**	Original Article
**Corresponding Author:**	Benjamin Wade, Ph.D.<br>University of California, Los Angeles<br>Los Angeles, CA UNITED STATES
**Corresponding Author Secondary Information:**	
**Corresponding Author's Institution:**	University of California, Los Angeles
**Corresponding Author's Secondary Institution:**	
**First Author:**	Benjamin Wade, Ph.D.
**First Author Secondary Information:**	
**Order of Authors:**	Benjamin Wade, Ph.D.
	Joana Loureiro
	Ashish Sahib
	Antoni Kubicki
	Shantanu Joshi
	Gerhard Hellemann
	Randall Espinoza
	Roger Woods
	Eliza Congdon
	Katherine Narr
**Order of Authors Secondary Information:**	
**Manuscript Region of Origin:**	UNITED STATES
**Abstract:**	Background: Ketamine is a rapidly-acting antidepressant treatment with robust response rates. Previous studies have reported that serial ketamine therapy modulates resting state functional connectivity in several large-scale networks, though it remains unknown whether variations in brain structure, function and connectivity impact subsequent treatment success. We used a data-driven approach to determine whether pretreatment multimodal neuroimaging measures predict changes along symptom dimensions of depression following serial ketamine infusion. Methods: Patients with depression (n=60) received structural, resting state functional and diffusion MRI scans before treatment. Depressive symptoms were assessed using the 17-item Hamilton Depression Rating Scale (HDRS-17), the Inventory of Depressive Symptomatology (IDS-C), and the Rumination Response Scale (RRS) before and 24h after patients received four (0.5mg/kg) infusions of racemic ketamine over 2-weeks. Nineteen unaffected controls were assessed at similar timepoints. Random forest regression (RFR) models predicted symptom changes using pretreatment multimodal neuroimaging and demographic measures. Results: Two HDRS-17 subscales, the HDRS-6 and core mood and anhedonia (CMA) symptoms, and the RRS: Reflection (RRSR) scale were predicted significantly with 19%, 27%, and 1% variance explained, respectively. Increased right medial prefrontal cortex (mPFC)/anterior cingulate (ACC)

Powered by Editorial Manager® and ProduXion Manager® from Aries Systems Corporation

	and posterior insula (PoI) and lower kurtosis of the superior longitudinal fasciculus predicted reduced HDRS-6 and CMA symptoms following treatment. RRSR change was predicted by global connectivity of the left posterior cingulate, left insula, and right superior parietal lobule. Conclusions: Our findings support that connectivity of the anterior default mode network and posterior insula may serve as potential biomarkers of antidepressant outcomes for core depressive symptoms.

Powered by Editorial Manager® and ProduXion Manager® from Aries Systems Corporation

Main Document (manuscript) Click here to access/download;Main Document (manuscript);Ketamine_Manuscript_PM.docx

Anterior Default Mode Network and Posterior Insular Connectivity is Predictive of Depressive Symptom Reduction Following Serial Ketamine Infusion

Benjamin S.C. Wade[1], Joana Loureiro[1], Ashish Sahib[1], Antoni Kubicki[1], Shantanu H. Joshi[1], Gerhard Hellemann[2], Randall T. Espinoza[2], Roger P. Woods[1,2], Eliza Congdon[2], Katherine L. Narr[1,2]

[1]Ahmanson-Lovelace Brain Mapping Center, Department of Neurology, University of California, Los Angeles, CA, [2]Department of Psychiatry and Biobehavioral Sciences, Semel Institute, UCLA, Los Angeles, USA

Corresponding author:

Name: Benjamin S.C. Wade

Email: Benjamin.SC.Wade@gmail.com

Address: 635 Charles Young Drive South Los Angeles, CA 90095

Phone number: 310-206-2101

Fax number: 310-206-5518

Word Count: 4500

Abstract

Background: Ketamine is a rapidly-acting antidepressant treatment with robust response rates. Previous studies have reported that serial ketamine therapy modulates resting state functional connectivity in several large-scale networks, though it remains unknown whether variations in brain structure, function and connectivity impact subsequent treatment success. We used a data-driven approach to determine whether pretreatment multimodal neuroimaging measures predict changes along symptom dimensions of depression following serial ketamine infusion. **Methods**: Patients with depression (n=60) received structural, resting state functional and diffusion MRI scans before treatment. Depressive symptoms were assessed using the 17-item Hamilton Depression Rating Scale (HDRS-17), the Inventory of Depressive Symptomatology (IDS-C), and the Rumination Response Scale (RRS) before and 24h after patients received four (0.5mg/kg) infusions of racemic ketamine over 2-weeks. Nineteen unaffected controls were assessed at similar timepoints. Random forest regression (RFR) models predicted symptom changes using pretreatment multimodal neuroimaging and demographic measures. **Results**: Two HDRS-17 subscales, the HDRS-6 and core mood and anhedonia (CMA) symptoms, and the RRS: Reflection (RRSR) scale were predicted significantly with 19%, 27%, and 1% variance explained, respectively. Increased right medial prefrontal cortex (mPFC)/anterior cingulate (ACC) and posterior insula (PoI) and lower kurtosis of the superior longitudinal fasciculus predicted reduced HDRS-6 and CMA symptoms following treatment. RRSR change was predicted by global connectivity of the left posterior cingulate, left insula, and right superior parietal lobule. **Conclusions**: Our findings support that connectivity of the anterior default mode network and posterior insula may serve as potential biomarkers of antidepressant outcomes for core depressive symptoms.

ClinicalTrials.gov: Biomarkers of Fast Acting Therapies in Major Depression, https:// clinicaltrials.gov/ct2/show/NCT02165449, NCT02165449

Introduction

Depression has a lifetime prevalence of over 20% (Hasin et al., 2018) making it a leading cause of disability. Compounding this, remission rates following common initial pharmaceutical interventions are about 30% with increasingly lower rates as patients fail to recover following subsequent interventions (Gaynes et al., 2009). An added bottleneck is the potential lag time between treatment initiation and the onset of antidepressant effect for most pharmaceutical and behavioral interventions. These circumstances stress the importance of identifying treatment-response biomarkers to serve as potential targets and understand mechanisms to guide more effective interventions, especially for rapidly-acting antidepressant treatments.

Ketamine is an N-methyl-d-aspartate (NMDA) receptor antagonist shown to induce robust and rapidly-acting antidepressant effects when administered in subanesthetic doses (R. M. Berman et al., 2000; Zarate Jr et al., 2006). Ketamine's antidepressant effects occur within hours and cumulatively over days (Aan Het Rot, Zarate, Charney, & Mathew, 2012). Though the antidepressant effects of a single intravenous ketamine treatment are short-lived, repeated infusions performed roughly 2-3 times weekly can prolong these effects to weeks and longer (James W Murrough et al., 2013; Shiroma et al., 2014; Singh et al., 2016). Ketamine is a relatively novel antidepressant treatment, thus its effects on neural structure or function remain under active investigation. Relatively few studies have specifically investigated treatment-response biomarkers. Despite different methodologies employed across studies, a recent qualitative review (McMillan & Muthukumaraswamy, 2020) highlights that ketamine infusion generally preserves or enhances cortico-subcortical connectivity patterns captured by resting state functional connectivity (RSFC) (Dandash et al., 2015; Grimm et al., 2015; Höflich et al., 2015), while corticocortical connectivity is widely disrupted (Bonhomme et al., 2016; Joules et al., 2015; Schroeder et al., 2016). A recent review by Alario & Niciu surveyed genetic, RSFC, neurophysiological predictors of response to ketamine. The authors reported that ketamine generally normalized disrupted functional connectivity in patients with MDD, though study-specific results have varied and have largely failed to be replicated. Despite this, connectivity of the insula, anterior cingulate, and left amygdala were widely reported in relation to response to ketamine (Alario & Niciu, 2021). A recent study by our group using Arterial Spin Labelling (ASL) suggests perfusion

of the bilateral hippocampus and insula is reduced with serial infusions (Sahib, Loureiro, Vasavada, Kubicki, Joshi, et al., 2020). A related study probing treatment-related changes in RSFC reported that ketamine normalized aberrant somatomotor and default mode network (DMN) connectivity. Serial infusions also reportedly reduce connectivity between the cerebellum and salience network (SN) (Sahib, Loureiro, Vasavada, Anderson, et al., 2020). Using a Go/No-Go task, we identified decreased functional activity in regions involved in inhibitory tasks including the DLPFC and inferior frontal cortex following serial ketamine treatments. To date, studies investigating predictors of clinical outcomes following ketamine infusion have been largely correlative rather than predictive. However, a pilot study by our group in an unrelated cohort found that symptom improvement 24-hours following a single infusion of ketamine was related to pretreatment white matter integrity of the cingulum and forceps minor (Vasavada et al., 2016). A study by Abdallah and colleagues reported more robust antidepressant effects of ketamine in patients with smaller pretreatment left hippocampal volumes 24-hours following treatment (Abdallah et al., 2015). In a task fMRI study, Murrough et al. reported that increased pretreatment connectivity of the right caudate while subjects were viewing positive emotional faces was associated with more reduced depressive symptoms following a single dose of intravenous ketamine (J W Murrough et al., 2015). Another study reported reduced connectivity between the lateral prefrontal cortex and subgenual anterior cingulate cortex associated with favorable antidepressant response following a single dose of ketamine (Gärtner et al., 2019). Another study identified that baseline cerebral blood flow (CBF) of the fusiform and visual cortex was related to single and serial infusion response likelihood, respectively (Sahib, Loureiro, Vasavada, Kubicki, Joshi, et al., 2020).

Although its multifaceted etiology is poorly understood, depression is widely characterized as a brain network disorder involving disrupted RSFC of large-scale resting state networks (RSNs) including the DMN, SN, and central executive network (CEN) (Kaiser, Andrews-Hanna, Wager, & Pizzagalli, 2015; Menon, 2011; Sheline, Price, Yan, & Mintun, 2010). Aberrant activity of these RSNs may be mediated in part by structural abnormalities of specific subregions or microstructural alterations of connective white matter. Converging evidence suggests that patients with depression exhibit increased connectivity within

the anterior DMN, increased connectivity between the SN and anterior DMN, altered connectivity between anterior and posterior DMN, and decreased connectivity between the CEN and posterior DMN (Mulders, van Eijndhoven, Schene, Beckmann, & Tendolkar, 2015).

Structural neuroimaging has highlighted reduced hippocampal volumes depressed patients (Schmaal et al., 2016). The hippocampus is broadly involved in learning and memory as well as emotional regulation processes relevant to depression (Gulyaeva, 2015). Due to its high concentration of glucocorticoid receptors, it is also highly prone to stress-related atrophy mediated by the release of circulating glucocorticoids by the hypothalamic-pituitary-adrenal axis (Sean M Smith & Vale, 2006). Also widely implicated in depression pathophysiology are structural abnormalities of the anterior cingulate, prefrontal cortex, and amygdala (Drevets, Savitz, & Trimble, 2008; Lorenzetti, Allen, Fornito, & Yücel, 2009; Schmaal et al., 2016). Numerous microstructural abnormalities of white matter tracts have also been identified in depression including the superior longitudinal fasciculus, uncinate fasciculus, corona radiata, and cingulum (Kieseppä et al., 2010; Korgaonkar et al., 2011; Murphy & Frodl, 2011; Wang, Leonards, Sterzer, & Ebinger, 2014). Whether these observations of structural and functional imaging abnormalities commonly presented in patients with depression predict antidepressant response to ketamine remains unknown.

Univariate, correlative studies have done much to advance our understanding of treatment-response biomarkers. However, robust biomarkers may lie within a multivariate space. Further, putative biomarkers should be validated using hold-out data. Thus, machine learning models trained and tested using rigorous cross validation offer a means by which to directly evaluate the predictive validity of potential multivariate biomarkers. The identification of biomarkers predictive of antidepressant outcomes following serial ketamine infusion (SKI) may be of substantial benefit for advancing the development of personalized treatment strategies for ketamine and potentially also for other fast-acting therapies. Here, we evaluated 60 patients with major depression undergoing a series of four ketamine infusions and 19 unaffected and untreated controls. We constructed purely data-driven machine learning models to predict individual reductions in depressive symptoms following treatment using pretreatment multimodal RSFC, structural

neuroimaging and demographic data. We hypothesized that pretreatment RSFC of the DMN and SN as well as measures of key limbic structures such as the hippocampus would be most informative of clinical outcomes following SKI.

Methods and Materials

Participants

Sixty patients experiencing a major depressive episode diagnosed by the Structured Clinical Interview for DSM-V were assessed between January 2017 and April 2020. Nineteen unaffected controls were included to determine whether ketamine significantly reduced symptoms compared to an untreated cohort. Table 1 details patient characteristics. Notably, participants overlap with previous studies (Loureiro et al., 2020; Sahib, Loureiro, Vasavada, Kubicki, Wade, et al., 2020; Sahib, Loureiro, Vasavada, Anderson, et al., 2020; Vasavada et al., 2020). Patient inclusion criteria included failure to respond adequately to at least two prior antidepressant medications, age between 20-64 years, DSM-5 diagnosis of major depression, a current episode of depression lasting for at least 6 months, HAM-D-17 scores ≥17, and stable antidepressant or mood stabilizer use (i.e., no treatment changes) for at least 6 weeks. Exclusionary criteria included rapid-cycling bipolar disorder, psychotic reactions to medication, intellectual disability or developmental disorders, comorbid substance abuse in the past three months, diagnosis of schizophrenia/schizoaffective disorder, Alzheimer's disease, or receipt of neuromodulation or ketamine treatment in the past 6 months. All patients underwent MRI scanning and clinical assessments within one week of their first treatment and again 24 hours following the end treatment (if the last treatment occurred on a Friday, assessments occurred 72 hours post treatment on a Monday). Depressive and ruminative symptoms were assessed before and after treatment using the 17-item Hamilton Depression Rating Scale (HDRS), Inventory of Depressive Symptomatology, 16-item Quick Inventory of Depressive Symptomatology Self Report (QIDS-SR), and the 10-item Rumination Response Scale (Treynor, Gonzalez, & Nolen-Hoeksema, 2003). Control participant symptoms were assessed twice approximately 2-3 weeks apart using the same scales (except the RRS). Inclusion criteria for non-depressed control participants were an age between 20-64 years, no history

of depressive disorder or bipolar disorder that is current, recurrent, or with a single episode that lasted longer than one year, no use antidepressants or mood stabilizers within the past 6 months, ability to read and understand English, and an ability to provide informed consent. Exclusionary criteria for controls included substance abuse in the past three months, diagnosis of schizophrenia/schizoaffective disorder, prior use of antidepressants mood stabilizers, developmental disorders, or a diagnosis of dementia. All participants received measurement of vital signs, a blood draw to determine metabolic, kidney and liver function, EKG, and provided a urine sample for drug and pregnancy (women only) screening. Drug and pregnancy screens were required to be negative, and all lab results were reviewed by the study physician to ensure there were no contraindications to participating in the study. All participants provided written informed consent following procedures approved by the UCLA Institutional Review Board (IRB).

SKI Treatment

Patients received a total of four ketamine infusions spaced 2 to 3 days apart. Ketamine was administered in subanesthetic doses (0.5 mg/kg) diluted in 60cc of saline and delivered intravenously via a pump over a 40-minute session at the UCLA Clinical and Translational Center Research (CTRC) or Resnick Neuropsychiatric Hospital. Vital signs including blood pressure, pulse oximetry, and respiratory rates were monitored during ketamine administration. Patients receiving ketamine were allowed to continue preapproved monoaminergic antidepressant medications, i.e., serotonin, norepinephrine, and dopamine reuptake inhibitors, and tricyclics; however, benzodiazepines were discontinued 24 hours prior to all scanning and treatment sessions.

Image Acquisition and Preprocessing

Images were acquired using a Siemens 3T Prisma MRI system at UCLA's Brain Mapping Center with a 32-channel phased array head coil. Acquisition sequences were identical to the Human Connectome Project Lifespan studies for Aging and Development (https://www.humanconnectome.org). Detailed acquisition

parameters, processing steps, and extraction of multimodal imaging measures are outlined in *Supplementary Methods*.

Predictive Features

Demographic (age and sex) and pretreatment multimodal imaging features were included as predictors. Multimodal imaging data were visually inspected and preprocessed using HCP minimal pipelines (Glasser et al., 2013; Stephen M Smith et al., 2013) with the BIDS-App (Gorgolewski et al., 2017). Functional imaging artifacts were removed using a modified spatial independent components analysis (sICA) method (Griffanti et al., 2014) and FSL's FIX (https://fsl.fmrib.ox.ac.uk/fsl/fslwiki/FIX). MSMall registration aligned cortical regions using measures of cortical folding, thickness, myelination, and resting-state connectivity information (Glasser et al., 2016; Robinson et al., 2018, 2014). Resting-state time series data was represented on the cortical surface and the time series was averaged across AP and PA acquisitions. Structural imaging features included regional estimates of cortical thickness in 68 regions based on the Desikan-Killiany atlas (Desikan et al., 2006), 24 subcortical volumes, diffusion measures (fractional anisotropy (FA), radial (RD), axial (AD), mean diffusivity (MD), and diffusion kurtosis (DK)) of 48 white matter tracts. NiLearn scripts were used to compute functional global connectivity measures of 360 cortical and 21 subcortical regions. Subjectwise correlation matrices were thresholded at $r \geq |0.3|$ to create a binary adjacency matrix for each subject. The node degree of each regional parcellation was computed as the number of other regions with which a given region was correlated above the threshold of $r \geq |0.3|$, providing a proxy of regional global connectivity. A tabulation of regional predictors is included in Table S1.

Clinical Outcome Measures

Depression is a symptomatically heterogenous disorder. The diversity of potential symptom profiles motivates the evaluation of treatment outcomes along multiple symptom dimensions. Thus, we constructed models to predict change along multiple scales: the 17- and 6-item HDRS (Bech et al., 1981), the IDS-C, the QIDS-SR, the brooding and rumination dimensions of the Rumination Response Scale (RRSB and

RRSR), and three symptom dimensions of the 17-item HDRS identified previously (Benjamin S C Wade et al., 2020, 2021): core mood and anhedonia (CMA), somatic disturbances (SOD), and insomnia. Subscales of the HDRS-17 used here are outlined in Table 2.

Predictive Modeling

We trained separate random forest regression (RFR) models, each with 5000 underlying regression trees, to predict change along each symptom set using pretreatment predictors outlined above. Models were trained and validated using 10-repeated 10-fold cross validation. The primary measure of model performance was the sums of squares formulation of the R^2; i.e., 1 - $\frac{\sum_i (y_i - \hat{y}_i)^2}{\sum_i (y_i - \bar{y})^2}$, where $\hat{y}_i$ and $\bar{y}_i$ are the predicted outcome and the i-th subject and the average outcome across all subjects in the testing folds, respectively (Poldrack, Huckins, & Varoquaux, 2020). We additionally report the normalized RMSE (NRMSE) value to facilitate comparisons across scales with different ranges: NRMSE = $\frac{RMSE}{y_{max} - y_{min}}$ where y_{max} and y_{min} are the maximum and minimum outcome values, respectively, in the test observations. The significance of each model's performance was assessed using permutation tests (100 resamples). We adjusted for multiple comparisons across the set of symptom sets using the standard FDR approach.

Results

Cohort Characteristics

Sixty patients (age=40.1 $\pm$11.1 years, n=30 males) were included. Patient age was associated with several outcome measures: the HDRS-6, QIDS-SR, IDS-C, and the SoD dimension (all p<0.05) with older patients showing a greater reduction in symptoms, on average, though sex was not significantly associated with outcomes. Using the HDRS-17 criterion of 50% symptom reduction, 37 (61%) patients experienced clinical response following SKI and 30 (50%) patients achieved remission (defined as a post-treatment HDRS-17 total score ≤7). We used unpaired, two-sample t-tests to compare the degree of symptom changes between

patients and controls. As expected, symptoms captured by the HDRS-17, IDS-C, and QIDS-SR, were significantly more reduced among patients than controls (all p<0.0001).

Model Performance

In patients, HDRS-6 and CMA change were predicted most accurately with R^2 scores of 0.19 (q<0.05) and 0.27 (q<0.05), respectively. The RRSR scale was predicted with a modest but significant R^2 score of 0.01 (q<0.05). No other symptom sets were predicted significantly above chance after adjustment for multiple comparisons. Model performance measures (R^2 and NRMSE) are outlined in **Figure 1** and Table 3, while plots of predicted versus actual symptom changes are illustrated in **Figure S1**.

Features Informative of Outcomes

The most informative features in the prediction of HDRS-6 and CMA changes were the global connectivity of the right posterior insular area 2 (PoI2) and the right Brodmann area (BA) 10r comprising the anterior cingulate cortex (ACC) and medial prefrontal cortex (mPFC), and the DK of the right superior longitudinal fasciculus (SLF). In **Figure 2** we show partial dependence plots (PDP) that illustrate the expected symptom changes for important predictors while holding all other predictors in the model at observed constants. These show that increased global connectivity of areas PoI2 and 10r predicted greater reduction in HDRS-6 and CMA symptoms while increased DK of the SLF predicted less reduction of HDRS-6 and CMA symptoms.

Change in the RRSR scale was predicted by the pretreatment global connectivity of the right BA 7PL (posterolateral BA 7; superior parietal cortex), the left insular gyrus, and left v23ab subdivision of the posterior cingulate cortex (PCC) and right putamen volume. Higher global connectivity of right BA 7PL, left insular gyrus, and left area v23ab along with higher right putamen volume predicted poorer reduction of ruminative symptoms.

Evaluation of Confounding Variables

We evaluated whether predictive imaging measures outlined above (PoI2, BA 10r, SLF DK, BA 7PL, left insular gyrus, and left v23ab) and significantly-predicted outcomes were associated with potential confounding clinical and demographic variables. Measures of global connectivity are based on counts, thus we evaluated associations with potential confounding variables using Poisson regression. The diffusivity of the SLF and thickness of the left insular gyrus were normally distributed (following a Shapiro-Wilk test; $p>0.05$) and continuous measures; thus, associations with confounds were evaluated using standard linear regression models. Global connectivity of the right PoI2 was associated with age, sex, education, duration of current episode, and current SSRI, SNRI, and benzodiazepine use (all $p<0.05$). Global connectivity of right BA 10r was associated with age, sex, duration of current episode, and current SNRI use (all $p<0.05$). Global connectivity of right BA 7PL was associated with sex, education, current episode duration, and current SSRI use (all $p<0.05$). Left area v23ab was associated with current episode duration, and current use of antipsychotics and SNRIs (all $p<0.05$). The kurtosis of the right SLF was not associated with the evaluated confounding variables. The left insula was associated with patient age and SSRI use (all $p<0.05$). We reran the original RFR models with all of the original predictors with the addition of medication use history, depressive episode duration, and education level. T-tests revealed no significant differences between original and updated model performance upon inclusion of additional confounding variables (all $p>0.9$). Further, no potential confounding clinical or demographic variables were among important predictors in the updated models. Thus, despite their associations with important imaging predictors, these potentially confounding variables did not improve prediction of outcomes for any dimension.

Discussion

We explored whether changes in depressive symptoms following SKI were predictable using multivariate patterns of pretreatment RSFC and demographic variables. Because depression is a symptomatically heterogenous disorder, we evaluated whether symptomatic changes were predictable across multiple depression scales and HDRS subscales. We observed a wide spread of performances across outcomes suggesting that changes along certain symptom clusters are more robustly predictable than others. In

particular, symptom changes along the HDRS-6 and Core Mood and Anhedonia symptom clusters were predicted most accurately. Notably, these are two subscales of the HDRS-17 with three overlapping items: depressed mood, work and activities, and psychomotor retardation. Similarly, change along these two scales was informed most by the pretreatment global connectivity of the right posterior insula 2 (PoI2) and BA 10r spanning the anterior cingulate and mPFC, and diffusivity of the SLF.

Right BA 10r encompasses portions of the ACC and mPFC; components of the anterior DMN. Previous depression studies have generally reported decreased connectivity of the anterior and posterior components of the DMN. For example, van Tol reported decreased connectivity of the dorsal mPFC with posterior portions of the DMN including the precuneus, angular gyrus, and middle temporal gyrus in depression relative to unaffected controls (van Tol et al., 2014). The same study reported elevated connectivity between the dorsal mPFC and components of the salience network: the insula and superior frontal gyrus. Several studies have investigated connectivity of the anterior DMN using independent components analysis (ICA). Among these, the anterior DMN has been consistently reported to be hyperconnected in patients relative to controls (Greicius et al., 2007; Mulders et al., 2015; Zhu et al., 2012). In related work, Abdallah et al. identified no differences in regional global connectivity between subsequent responders and non-responders to single-infusion ketamine, however, ketamine responders showed increased change in connectivity of the right lateral PFC with treatment (Abdallah et al., 2017). Notably, a previous study showed that RSFC between the pregenual ACC and right lateral PFC was significantly associated with the extent of symptom reduction 24h after single ketamine infusion therapy (racemic and S-ketamine) in major depression in line with our findings (Gärtner et al., 2019). A double-blind, placebo-controlled, crossover study reported that connectivity between the insula and DMN was normalized compared to controls two days following a single ketamine infusion (Evans et al., 2018), further implicating regions identified here. A combined EEG/fMRI study by Zacharias in healthy volunteers reported that ketamine reduced connectivity of the anterior DMN (Zacharias et al., 2020). Similarly, Li and colleagues reported that a single infusion of ketamine reduced connectivity between the posterior cingulate cortex and the dorsomedial prefrontal cortex in 61 healthy participants (Li et al., 2020). Apart from ketamine, RSFC

of anterior DMN components have been reported to be predictive of antidepressant response to first-line antidepressant medications (Kozel et al., 2011), cognitive behavioral therapy (Dunlop et al., 2017), transcranial magnetic stimulation (Fox, Buckner, White, Greicius, & Pascual-Leone, 2012; Salomons et al., 2014), and electroconvulsive therapy (van Waarde et al., 2015).

The right PoI2 is a subdivision of the posterior insula and frontal opercular complex and is a component of the ventral attention network DMN and the externally directed CEN, the functional role of the posterior insula has largely been ascribed to sensorimotor tasks with affective significance (Craig, 2009; Kurth, Zilles, Fox, Laird, & Eickhoff, 2010). Hu et al. used amplitude of low-frequency fluctuation (ALFF) analysis investigate intrinsic neural oscillation abnormalities of the insula in adolescence with depression and reported decreased activity of the right posterior insula in youth with depression relative to unaffected controls (Hu et al., 2019). A follow-up seed-based analysis in the same cohort revealed reduced functional connectivity between the right posterior insula and several visual, somatomotor, and limbic regions (Hu et al., 2019). In a cohort of 19 patients with depression and 19 controls, Peng et al. reported on decreased functional connectivity between the right posterior insula and the posterior parietal cortex, part of the CEN, in patients relative to controls (Peng et al., 2018). Ketamine has also been shown to increase the global connectivity of the insula in responsive patients (Abdallah et al., 2017). An open-label Magnetoencephalography study of treatment-related effects of ketamine reported a reduction in connectivity between an insulo-temporal ICA component and the amygdala (Nugent, Robinson, Coppola, & Zarate Jr, 2016). In an overlapping sample, using positron emission tomography, the same group reported decreased metabolism of the insula following a single dose of ketamine (Carlson et al., 2013). These findings support that the insula may mediate specific symptom expressions in depression while our current findings support that disruptions in these same regions may be related to treatment response likelihood. Indeed, one study has shown RSFC between the pregenual ACC and insula predict differential outcomes following treatment with medication and cognitive behavioral therapy in major depression (Dunlop et al., 2017).

The pretreatment DK of the superior longitudinal fasciculus (SLF) diffusion was predictive of both HDRS-6 and CMA changes. The SLF has widely been associated with depressive symptoms (de Diego-Adeliño et al., 2014; Jiang et al., 2017; Korgaonkar et al., 2011; Reppermund et al., 2014) and in several studies, fractional anisotropy (FA) of this tract was found inversely correlated with the severity of depressive symptoms (Lai & Wu, 2014) and illness duration (de Diego-Adeliño et al., 2014; Gu et al., 2013). A recent meta-analysis of microstructural abnormalities in medication-free patients with depression identified robust FA reductions in the SLF III (Jiang et al., 2017), which connects the supramarginal gyrus and ventral premotor and prefrontal cortices. The connectivity of this tract suggests that it plays a role in processing somatosensory information (Makris et al., 2005). As psychomotor retardation is a symptom captured both by the HDRS-6 and CMA symptom dimensions this offers a plausible link between the SLF and its role as a potential biomarker of symptom reduction in these related dimensions. Increased DK reflects an increasingly complex microstructural environment, implying more obstacles to normal diffusion (Steven, Zhuo, & Melhem, 2013), such as increased cell density or complexity, while lower DK may suggest atrophied cellular structure. Here, we observed more reduced HDRS-6 and CMA symptoms among patients with lower DK in the right SLF that may suggest that depression-related microstructural abnormalities of the SLF is indicative of subsequent responsivity to SKI. Related to these findings, a pilot study on thirteen patients with treatment-resistant depression who received a single infusion of ketamine reported that higher FA of the left SLF was associated with better symptom improvement 24-hours post-infusion (Sydnor et al., 2020).

Ruminative symptoms are commonly described by a two-factor model with subcomponents of brooding and reflection. Brooding has been more consistently linked with maladaptive cognitive traits of depression (Lo, Ho, & Hollon, 2008) and suicide ideation (Surrence, Miranda, Marroquín, & Chan, 2009); however, reflective symptoms are related to depression severity (Fresco, Frankel, Mennin, Turk, & Heimberg, 2002; Surrence et al., 2009). Here, change in reflective (RRSR) symptoms were predicted significantly. In a sample of 10 patients with treatment-resistant depression, Vidal et al. reported that a single infusion of ketamine was effective at reducing ruminative symptoms (Vidal, Jermann, Aubry,

Richard-Lepouriel, & Kosel, 2020). Here, we saw that change in RRSR symptoms was predicted by pretreatment global connectivity of left ventral area 23ab (in the PCC), the left insular granular complex, area 7PL of the right superior parietal cortex, and the volume of the right putamen. Increased RSFC and volume was associated with less reduction of RRSR symptoms. The PCC is a hub of the DMN and hyperconnectivity of this region has widely been associated with increased ruminative symptoms (M. G. Berman et al., 2011; Cooney, Joormann, Eugène, Dennis, & Gotlib, 2010). This may suggest that increased connectivity of the PCC along with components of the dorsal attention (superior parietal cortex) and somatomotor networks (insular granular complex) may reflect increased treatment resistance to ketamine.

Lastly, it was hypothesized that hippocampal volume or connectivity would be a predictor of response to SKI given that it has been associated with response to other treatments including electroconvulsive therapy (B.S.C. Wade et al., 2017) and pharmaceutical treatments (Colle et al., 2018). Further, the effects of depression on hippocampal structure have been widely reported (Sheline, Wang, Gado, Csernansky, & Vannier, 1996) making its structural or functional properties a plausible candidate biomarker of treatment response. Contrary to our expectations, however, hippocampal structure and function was not implicated as a predictor of response to SKI in this multivariate framework. It is possible that this may reflect distinct mechanisms of actions for SKI or a predominating association of more salient predictors in a multivariate framework that overshadow the contributions of the hippocampus.

Limitations

There are several limitations to consider in interpreting the findings of this study. Statistical and machine learning models are prone to overfitting to training data when the number of possible predictors exceeds the number of samples used to train the data. However, we used a conservative 10-repeated 10-fold cross validation approach to directly evaluate model performance in hold-out data. Further, this was not a randomized clinical trial and lacked an active placebo group. Additional considerations include that patients were allowed to continue on current and stable antidepressant medication treatments, however, these were not associated with key predictors or outcomes.

Conclusions

Using a purely data-driven approach and multimodal MRI, our study supports that pretreatment global connectivity of the ACC, mPFC, and posterior insula as well as diffusivity of the SLF are potential biomarkers of antidepressant outcomes following SKI. Importantly, these regions form nodes or structural connections encompassing the DMN and SN/VAN, and have been widely implicated in the pathology of depression thus adding to their plausibility in this role. We evaluated biomarkers of response along a number of widely-used symptom scales, sub-scales, and less commonly-used sub-scales identified by previous studies. We found that core symptoms of depression captured by the HDRS-6 and an overlapping dimension of CMA were predicted most accurately. Future work will investigate biomarkers of durable, long-term response to SKI. This work may advance the ultimate goal of using imaging or other physiological markers to personalize treatments to improve and speed recovery in individual patients.

Financial Support

This work is supported in part by a NARSAD Young Investigator Grant (27786 to BW) and a K99 Pathway to Independence Award (MH119314 to BW). Other investigators were supported by National Institute of Mental Health grants (1U01MH110008-01, MH092301, and MH102743), and the Muriel Harris Chair in Geriatric Psychiatry [to R.E.]).

Conflict of Interest

The authors report no conflicts of interest.

Ethical Standards

The authors assert that all procedures contributing to this work comply with the ethical standards of the relevant national and institutional committees on human experimentation and with the Helsinki Declaration of 1975, as revised in 2008

References

Aan Het Rot, M., Zarate, C. A. J., Charney, D. S., & Mathew, S. J. (2012). Ketamine for depression: where do we go from here? *Biological Psychiatry*, *72*(7), 537–547. https://doi.org/10.1016/j.biopsych.2012.05.003

Abdallah, C. G., Averill, L. A., Collins, K. A., Geha, P., Schwartz, J., Averill, C., … Murrough, J. W. (2017). Ketamine Treatment and Global Brain Connectivity in Major Depression. *Neuropsychopharmacology : Official Publication of the American College of Neuropsychopharmacology*, *42*(6), 1210–1219. https://doi.org/10.1038/npp.2016.186

Abdallah, C. G., Salas, R., Jackowski, A., Baldwin, P., Sato, J. R., & Mathew, S. J. (2015). Hippocampal volume and the rapid antidepressant effect of ketamine. *Journal of Psychopharmacology (Oxford, England)*, *29*(5), 591–595. https://doi.org/10.1177/0269881114544776

Alario, A. A., & Niciu, M. J. (2021). Biomarkers of ketamine's antidepressant effect: a clinical review of genetics, functional connectivity, and neurophysiology. *Chronic Stress*, *5*. https://doi.org/10.1177/24705470211014210

Bech, P., Allerup, P., Gram, L. F., Reisby, N., Rosenberg, R., Jacobsen, O., & Nagy, A. (1981). The Hamilton Depression Scale. *Acta Psychiatrica Scandinavica*, *63*(3), 290–299. https://doi.org/10.1111/j.1600-0447.1981.tb00676.x

Berman, M. G., Peltier, S., Nee, D. E., Kross, E., Deldin, P. J., & Jonides, J. (2011). Depression, rumination and the default network. *Social Cognitive and Affective Neuroscience*, *6*(5), 548–555. https://doi.org/10.1093/scan/nsq080

Berman, R. M., Cappiello, A., Anand, A., Oren, D. A., Heninger, G. R., Charney, D. S., & Krystal, J. H. (2000). Antidepressant effects of ketamine in depressed patients. *Biological Psychiatry*, *47*(4), 351–354. https://doi.org/10.1016/s0006-3223(99)00230-9

Bonhomme, V., Vanhaudenhuyse, A., Demertzi, A., Bruno, M.-A., Jaquet, O., Bahri, M. A., … Laureys, S. (2016). Resting-state Network-specific Breakdown of Functional Connectivity during Ketamine Alteration of Consciousness in Volunteers. *Anesthesiology*, *125*(5), 873–888. https://doi.org/10.1097/ALN.0000000000001275

Carlson, P. J., Diazgranados, N., Nugent, A. C., Ibrahim, L., Luckenbaugh, D. A., Brutsche, N., … Drevets, W. C. (2013). Neural correlates of rapid antidepressant response to ketamine in treatment-resistant unipolar depression: a preliminary positron emission tomography study. *Biological Psychiatry*, *73*(12), 1213–1221. https://doi.org/10.1016/j.biopsych.2013.02.008

Colle, R., Dupong, I., Colliot, O., Deflesselle, E., Hardy, P., Falissard, B., … Corruble, E. (2018). Smaller hippocampal volumes predict lower antidepressant response/remission rates in depressed patients: A meta-analysis. *The World Journal of Biological Psychiatry : The Official Journal of the World Federation of Societies of Biological Psychiatry*, *19*(5), 360–367. https://doi.org/10.1080/15622975.2016.1208840

Cooney, R. E., Joormann, J., Eugène, F., Dennis, E. L., & Gotlib, I. H. (2010). Neural correlates of rumination in depression. *Cognitive, Affective & Behavioral Neuroscience*, *10*(4), 470–478. https://doi.org/10.3758/CABN.10.4.470

Craig, A. D. B. (2009, January). How do you feel--now? The anterior insula and human awareness. *Nature Reviews. Neuroscience*, Vol. 10, pp. 59–70. https://doi.org/10.1038/nrn2555

Dandash, O., Harrison, B. J., Adapa, R., Gaillard, R., Giorlando, F., Wood, S. J., … Fornito, A. (2015). Selective augmentation of striatal functional connectivity following NMDA receptor antagonism: implications for psychosis. *Neuropsychopharmacology : Official Publication of the American College of Neuropsychopharmacology*, *40*(3), 622–631. https://doi.org/10.1038/npp.2014.210

de Diego-Adeliño, J., Pires, P., Gómez-Ansón, B., Serra-Blasco, M., Vives-Gilabert, Y., Puigdemont, D., … Portella, M. J. (2014). Microstructural white-matter abnormalities associated with treatment resistance, severity and duration of illness in major depression. *Psychological Medicine*, *44*(6), 1171–1182. https://doi.org/10.1017/S003329171300158X

Desikan, R. S., Ségonne, F., Fischl, B., Quinn, B. T., Dickerson, B. C., Blacker, D., … Killiany, R. J. (2006). An automated labeling system for subdividing the human cerebral cortex on MRI scans into gyral based regions of interest. *NeuroImage*, *31*(3), 968–980. https://doi.org/10.1016/j.neuroimage.2006.01.021

Drevets, W. C., Savitz, J., & Trimble, M. (2008). The subgenual anterior cingulate cortex in mood disorders. *CNS Spectrums*, *13*(8), 663–681. https://doi.org/10.1017/S1092852900013754

Dunlop, B. W., Rajendra, J. K., Craighead, W. E., Kelley, M. E., McGrath, C. L., Choi, K. S., … Mayberg, H. S. (2017). Functional Connectivity of the Subcallosal Cingulate Cortex And Differential Outcomes to Treatment With Cognitive-Behavioral Therapy or Antidepressant Medication for Major Depressive Disorder. *The American Journal of Psychiatry*, *174*(6), 533–545. https://doi.org/10.1176/appi.ajp.2016.16050518

Evans, J. W., Szczepanik, J., Brutsché, N., Park, L. T., Nugent, A. C., & Zarate, C. A. J. (2018). Default Mode Connectivity in Major Depressive Disorder Measured Up to 10 Days After Ketamine Administration. *Biological Psychiatry*, *84*(8), 582–590. https://doi.org/10.1016/j.biopsych.2018.01.027

Fox, M. D., Buckner, R. L., White, M. P., Greicius, M. D., & Pascual-Leone, A. (2012). Efficacy of

transcranial magnetic stimulation targets for depression is related to intrinsic functional connectivity with the subgenual cingulate. *Biological Psychiatry*, *72*(7), 595–603. https://doi.org/10.1016/j.biopsych.2012.04.028

Fresco, D. M., Frankel, A. N., Mennin, D. S., Turk, C. L., & Heimberg, R. G. (2002). Distinct and Overlapping Features of Rumination and Worry: The Relationship of Cognitive Production to Negative Affective States. *Cognitive Therapy and Research*, *26*(2), 179–188. https://doi.org/10.1023/A:1014517718949

Gärtner, M., Aust, S., Bajbouj, M., Fan, Y., Wingenfeld, K., Otte, C., … Scheidegger, M. (2019). Functional connectivity between prefrontal cortex and subgenual cingulate predicts antidepressant effects of ketamine. *European Neuropsychopharmacology*, *29*(4), 501–508. https://doi.org/https://doi.org/10.1016/j.euroneuro.2019.02.008

Gaynes, B. N., Warden, D., Trivedi, M. H., Wisniewski, S. R., Fava, M., & Rush, A. J. (2009). What did STAR*D teach us? Results from a large-scale, practical, clinical trial for patients with depression. *Psychiatric Services (Washington, D.C.)*, *60*(11), 1439–1445. https://doi.org/10.1176/ps.2009.60.11.1439

Glasser, M. F., Coalson, T. S., Robinson, E. C., Hacker, C. D., Harwell, J., Yacoub, E., … Van Essen, D. C. (2016). A multi-modal parcellation of human cerebral cortex. *Nature*, *536*(7615), 171–178. https://doi.org/10.1038/nature18933

Glasser, M. F., Sotiropoulos, S. N., Wilson, J. A., Coalson, T. S., Fischl, B., Andersson, J. L., … Jenkinson, M. (2013). The minimal preprocessing pipelines for the Human Connectome Project. *NeuroImage*, *80*, 105–124. https://doi.org/10.1016/j.neuroimage.2013.04.127

Gorgolewski, K. J., Alfaro-Almagro, F., Auer, T., Bellec, P., Capotă, M., Chakravarty, M. M., … Poldrack, R. A. (2017). BIDS apps: Improving ease of use, accessibility, and reproducibility of neuroimaging data analysis methods. *PLoS Computational Biology*, *13*(3), e1005209–e1005209. https://doi.org/10.1371/journal.pcbi.1005209

Greicius, M. D., Flores, B. H., Menon, V., Glover, G. H., Solvason, H. B., Kenna, H., … Schatzberg, A. F. (2007). Resting-state functional connectivity in major depression: abnormally increased contributions from subgenual cingulate cortex and thalamus. *Biological Psychiatry*, *62*(5), 429–437. https://doi.org/10.1016/j.biopsych.2006.09.020

Griffanti, L., Salimi-Khorshidi, G., Beckmann, C. F., Auerbach, E. J., Douaud, G., Sexton, C. E., … Smith, S. M. (2014). ICA-based artefact removal and accelerated fMRI acquisition for improved resting state network imaging. *NeuroImage*, *95*, 232–247. https://doi.org/10.1016/j.neuroimage.2014.03.034

Grimm, O., Gass, N., Weber-Fahr, W., Sartorius, A., Schenker, E., Spedding, M., … Meyer-Lindenberg,

A. (2015). Acute ketamine challenge increases resting state prefrontal-hippocampal connectivity in both humans and rats. *Psychopharmacology*, *232*(21–22), 4231–4241. https://doi.org/10.1007/s00213-015-4022-y

Gu, L., Xie, J., Long, J., Chen, Q., Chen, Q., Pan, R., … Su, L. (2013). Epidemiology of major depressive disorder in mainland china: a systematic review. *PloS One*, *8*(6), e65356. https://doi.org/10.1371/journal.pone.0065356

Gulyaeva, N. V. (2015). Ventral hippocampus, Stress and phychopathology: Translational implications. *Neurochemical Journal*, *9*(2), 85–94. https://doi.org/10.1134/S1819712415020075

Hasin, D. S., Sarvet, A. L., Meyers, J. L., Saha, T. D., Ruan, W. J., Stohl, M., & Grant, B. F. (2018). Epidemiology of Adult DSM-5 Major Depressive Disorder and Its Specifiers in the United States. *JAMA Psychiatry*, *75*(4), 336–346. https://doi.org/10.1001/jamapsychiatry.2017.4602

Höflich, A., Hahn, A., Küblböck, M., Kranz, G. S., Vanicek, T., Windischberger, C., … Lanzenberger, R. (2015). Ketamine-Induced Modulation of the Thalamo-Cortical Network in Healthy Volunteers As a Model for Schizophrenia. *The International Journal of Neuropsychopharmacology*, *18*(9). https://doi.org/10.1093/ijnp/pyv040

Hu, L., Xiao, M., Ai, M., Wang, W., Chen, J., Tan, Z., … Kuang, L. (2019). Disruption of resting-state functional connectivity of right posterior insula in adolescents and young adults with major depressive disorder. *Journal of Affective Disorders*, *257*, 23–30. https://doi.org/10.1016/j.jad.2019.06.057

Jiang, J., Zhao, Y.-J., Hu, X.-Y., Du, M.-Y., Chen, Z.-Q., Wu, M., … Gong, Q.-Y. (2017). Microstructural brain abnormalities in medication-free patients with major depressive disorder: a systematic review and meta-analysis of diffusion tensor imaging. *Journal of Psychiatry & Neuroscience : JPN*, *42*(3), 150–163. https://doi.org/10.1503/jpn.150341

Joules, R., Doyle, O. M., Schwarz, A. J., O'Daly, O. G., Brammer, M., Williams, S. C., & Mehta, M. A. (2015). Ketamine induces a robust whole-brain connectivity pattern that can be differentially modulated by drugs of different mechanism and clinical profile. *Psychopharmacology*, *232*(21–22), 4205–4218. https://doi.org/10.1007/s00213-015-3951-9

Kaiser, R. H., Andrews-Hanna, J. R., Wager, T. D., & Pizzagalli, D. A. (2015). Large-Scale Network Dysfunction in Major Depressive Disorder: A Meta-analysis of Resting-State Functional Connectivity. *JAMA Psychiatry*, *72*(6), 603–611. https://doi.org/10.1001/jamapsychiatry.2015.0071

Kieseppä, T., Eerola, M., Mäntylä, R., Neuvonen, T., Poutanen, V.-P., Luoma, K., … Isometsä, E. (2010). Major depressive disorder and white matter abnormalities: a diffusion tensor imaging study with tract-based spatial statistics. *Journal of Affective Disorders*, *120*(1–3), 240–244. https://doi.org/10.1016/j.jad.2009.04.023

Korgaonkar, M. S., Grieve, S. M., Koslow, S. H., Gabrieli, J. D. E., Gordon, E., & Williams, L. M. (2011). Loss of white matter integrity in major depressive disorder: evidence using tract-based spatial statistical analysis of diffusion tensor imaging. *Human Brain Mapping*, *32*(12), 2161–2171. https://doi.org/10.1002/hbm.21178

Kozel, F. A., Rao, U., Lu, H., Nakonezny, P. A., Grannemann, B., McGregor, T., … Trivedi, M. H. (2011). Functional connectivity of brain structures correlates with treatment outcome in major depressive disorder. *Frontiers in Psychiatry*, *2*, 7. https://doi.org/10.3389/fpsyt.2011.00007

Kurth, F., Zilles, K., Fox, P. T., Laird, A. R., & Eickhoff, S. B. (2010). A link between the systems: functional differentiation and integration within the human insula revealed by meta-analysis. *Brain Structure & Function*, *214*(5–6), 519–534. https://doi.org/10.1007/s00429-010-0255-z

Lai, C. H., & Wu, Y. T. (2014). Alterations in white matter micro-integrity of the superior longitudinal fasciculus and anterior thalamic radiation of young adult patients with depression. *Psychological Medicine*, *44*(13), 2825–2832. https://doi.org/10.1017/S0033291714000440

Li, M., Woelfer, M., Colic, L., Safron, A., Chang, C., Heinze, H.-J., … Walter, M. (2020). Default mode network connectivity change corresponds to ketamine's delayed glutamatergic effects. *European Archives of Psychiatry and Clinical Neuroscience*, *270*(2), 207–216. https://doi.org/10.1007/s00406-018-0942-y

Lo, C. S. L., Ho, S. M. Y., & Hollon, S. D. (2008). The effects of rumination and negative cognitive styles on depression: a mediation analysis. *Behaviour Research and Therapy*, *46*(4), 487–495. https://doi.org/10.1016/j.brat.2008.01.013

Lorenzetti, V., Allen, N. B., Fornito, A., & Yücel, M. (2009). Structural brain abnormalities in major depressive disorder: a selective review of recent MRI studies. *Journal of Affective Disorders*, *117*(1–2), 1–17. https://doi.org/10.1016/j.jad.2008.11.021

Loureiro, J. R. A., Leaver, A., Vasavada, M., Sahib, A. K., Kubicki, A., Joshi, S., … Narr, K. L. (2020). Modulation of amygdala reactivity following rapidly acting interventions for major depression. *Human Brain Mapping*, *41*(7), 1699–1710. https://doi.org/10.1002/hbm.24895

Makris, N., Kennedy, D. N., McInerney, S., Sorensen, A. G., Wang, R., Caviness, V. S. J., & Pandya, D. N. (2005). Segmentation of subcomponents within the superior longitudinal fascicle in humans: a quantitative, in vivo, DT-MRI study. *Cerebral Cortex (New York, N.Y. : 1991)*, *15*(6), 854–869. https://doi.org/10.1093/cercor/bhh186

McMillan, R., & Muthukumaraswamy, S. D. (2020). The neurophysiology of ketamine: an integrative review. *Reviews in the Neurosciences*. https://doi.org/10.1515/revneuro-2019-0090

Menon, V. (2011). Large-scale brain networks and psychopathology: a unifying triple network model. *Trends in Cognitive Sciences*, *15*(10), 483–506. https://doi.org/10.1016/j.tics.2011.08.003

Mulders, P. C., van Eijndhoven, P. F., Schene, A. H., Beckmann, C. F., & Tendolkar, I. (2015). Resting-state functional connectivity in major depressive disorder: A review. *Neuroscience and Biobehavioral Reviews*, *56*, 330–344. https://doi.org/10.1016/j.neubiorev.2015.07.014

Murphy, M. L., & Frodl, T. (2011). Meta-analysis of diffusion tensor imaging studies shows altered fractional anisotropy occurring in distinct brain areas in association with depression. *Biology of Mood & Anxiety Disorders*, *1*(1), 3. https://doi.org/10.1186/2045-5380-1-3

Murrough, J W, Collins, K. A., Fields, J., DeWilde, K. E., Phillips, M. L., Mathew, S. J., … Iosifescu, D. V. (2015). Regulation of neural responses to emotion perception by ketamine in individuals with treatment-resistant major depressive disorder. *Translational Psychiatry*, *5*(2), e509. https://doi.org/10.1038/tp.2015.10

Murrough, James W, Iosifescu, D. V, Chang, L. C., Al Jurdi, R. K., Green, C. E., Perez, A. M., … Mathew, S. J. (2013). Antidepressant efficacy of ketamine in treatment-resistant major depression: a two-site randomized controlled trial. *The American Journal of Psychiatry*, *170*(10), 1134–1142. https://doi.org/10.1176/appi.ajp.2013.13030392

Nugent, A. C., Robinson, S. E., Coppola, R., & Zarate Jr, C. A. (2016). Preliminary differences in resting state MEG functional connectivity pre- and post-ketamine in major depressive disorder. *Psychiatry Research. Neuroimaging*, *254*, 56–66. https://doi.org/10.1016/j.pscychresns.2016.06.006

Peng, X., Lin, P., Wu, X., Gong, R., Yang, R., & Wang, J. (2018). Insular subdivisions functional connectivity dysfunction within major depressive disorder. *Journal of Affective Disorders*, *227*, 280–288. https://doi.org/10.1016/j.jad.2017.11.018

Poldrack, R. A., Huckins, G., & Varoquaux, G. (2020). Establishment of Best Practices for Evidence for Prediction: A Review. *JAMA Psychiatry*, *77*(5), 534–540. https://doi.org/10.1001/jamapsychiatry.2019.3671

Reppermund, S., Zhuang, L., Wen, W., Slavin, M. J., Trollor, J. N., Brodaty, H., & Sachdev, P. S. (2014). White matter integrity and late-life depression in community-dwelling individuals: diffusion tensor imaging study using tract-based spatial statistics. *The British Journal of Psychiatry : The Journal of Mental Science*, *205*(4), 315–320. https://doi.org/10.1192/bjp.bp.113.142109

Robinson, E. C., Garcia, K., Glasser, M. F., Chen, Z., Coalson, T. S., Makropoulos, A., … Rueckert, D. (2018). Multimodal surface matching with higher-order smoothness constraints. *NeuroImage*, *167*, 453–465. https://doi.org/10.1016/j.neuroimage.2017.10.037

Robinson, E. C., Jbabdi, S., Glasser, M. F., Andersson, J., Burgess, G. C., Harms, M. P., … Jenkinson, M. (2014). MSM: A new flexible framework for multimodal surface matching. *NeuroImage*, *100*, 414–426. https://doi.org/10.1016/j.neuroimage.2014.05.069

Sahib, A. K., Loureiro, J. R. A., Vasavada, M. M., Kubicki, A., Joshi, S. H., Wang, K., … Narr, K. L. (2020). Single and repeated ketamine treatment induces perfusion changes in sensory and limbic networks in major depressive disorder. *European Neuropsychopharmacology : The Journal of the European College of Neuropsychopharmacology*, *33*, 89–100. https://doi.org/10.1016/j.euroneuro.2020.01.017

Sahib, A. K., Loureiro, J. R. A., Vasavada, M. M., Kubicki, A., Wade, B., Joshi, S. H., … Narr, K. L. (2020). Modulation of inhibitory control networks relate to clinical response following ketamine therapy in major depression. *Translational Psychiatry*, *10*(1), 260. https://doi.org/10.1038/s41398-020-00947-7

Sahib, A. K., Loureiro, J. R., Vasavada, M., Anderson, C., Kubicki, A., Wade, B., … Narr, K. L. (2020). Modulation of the functional connectome in major depressive disorder by ketamine therapy. *Psychological Medicine*, 1–10. https://doi.org/10.1017/S0033291720004560

Salomons, T. V, Dunlop, K., Kennedy, S. H., Flint, A., Geraci, J., Giacobbe, P., & Downar, J. (2014). Resting-state cortico-thalamic-striatal connectivity predicts response to dorsomedial prefrontal rTMS in major depressive disorder. *Neuropsychopharmacology : Official Publication of the American College of Neuropsychopharmacology*, *39*(2), 488–498. https://doi.org/10.1038/npp.2013.222

Schmaal, L., Veltman, D. J., Van Erp, T. G. M., Smann, P. G., Frodl, T., Jahanshad, N., … Hibar, D. P. (2016). Subcortical brain alterations in major depressive disorder: Findings from the ENIGMA Major Depressive Disorder working group. *Molecular Psychiatry*, *21*(6), 806–812. https://doi.org/10.1038/mp.2015.69

Schroeder, K. E., Irwin, Z. T., Gaidica, M., Nicole Bentley, J., Patil, P. G., Mashour, G. A., & Chestek, C. A. (2016). Disruption of corticocortical information transfer during ketamine anesthesia in the primate brain. *NeuroImage*, *134*, 459–465. https://doi.org/10.1016/j.neuroimage.2016.04.039

Sheline, Y. I., Price, J. L., Yan, Z., & Mintun, M. A. (2010). Resting-state functional MRI in depression unmasks increased connectivity between networks via the dorsal nexus. *Proceedings of the National Academy of Sciences of the United States of America*, *107*(24), 11020–11025. https://doi.org/10.1073/pnas.1000446107

Sheline, Y. I., Wang, P. W., Gado, M. H., Csernansky, J. G., & Vannier, M. W. (1996). Hippocampal atrophy in recurrent major depression. *Proceedings of the National Academy of Sciences of the United States of America*, *93*(9), 3908–3913. https://doi.org/10.1073/pnas.93.9.3908

Shiroma, P. R., Johns, B., Kuskowski, M., Wels, J., Thuras, P., Albott, C. S., & Lim, K. O. (2014). Augmentation of response and remission to serial intravenous subanesthetic ketamine in treatment resistant depression. *Journal of Affective Disorders*, *155*, 123–129. https://doi.org/10.1016/j.jad.2013.10.036

Singh, J. B., Fedgchin, M., Daly, E. J., De Boer, P., Cooper, K., Lim, P., … Van Nueten, L. (2016). A Double-Blind, Randomized, Placebo-Controlled, Dose-Frequency Study of Intravenous Ketamine in Patients With Treatment-Resistant Depression. *The American Journal of Psychiatry*, *173*(8), 816–826. https://doi.org/10.1176/appi.ajp.2016.16010037

Smith, Sean M, & Vale, W. W. (2006). The role of the hypothalamic-pituitary-adrenal axis in neuroendocrine responses to stress. *Dialogues in Clinical Neuroscience*, *8*(4), 383–395. https://doi.org/10.31887/DCNS.2006.8.4/ssmith

Smith, Stephen M, Beckmann, C. F., Andersson, J., Auerbach, E. J., Bijsterbosch, J., Douaud, G., … Glasser, M. F. (2013). Resting-state fMRI in the Human Connectome Project. *NeuroImage*, *80*, 144–168. https://doi.org/10.1016/j.neuroimage.2013.05.039

Steven, A. J., Zhuo, J., & Melhem, E. R. (2013). Diffusion Kurtosis Imaging: An Emerging Technique for Evaluating the Microstructural Environment of the Brain. *American Journal of Roentgenology*, *202*(1), W26–W33. https://doi.org/10.2214/AJR.13.11365

Surrence, K., Miranda, R., Marroquín, B. M., & Chan, S. (2009). Brooding and reflective rumination among suicide attempters: cognitive vulnerability to suicidal ideation. *Behaviour Research and Therapy*, *47*(9), 803–808. https://doi.org/10.1016/j.brat.2009.06.001

Sydnor, V. J., Lyall, A. E., Cetin-Karayumak, S., Cheung, J. C., Felicione, J. M., Akeju, O., … Kubicki, M. (2020). Studying pre-treatment and ketamine-induced changes in white matter microstructure in the context of ketamine's antidepressant effects. *Translational Psychiatry*, *10*(1). https://doi.org/10.1038/s41398-020-01122-8

Treynor, W., Gonzalez, R., & Nolen-Hoeksema, S. (2003). Rumination Reconsidered: A Psychometric Analysis. *Cognitive Therapy and Research*, *27*(3), 247–259. https://doi.org/10.1023/A:1023910315561

van Tol, M.-J., Li, M., Metzger, C. D., Hailla, N., Horn, D. I., Li, W., … Walter, M. (2014). Local cortical thinning links to resting-state disconnectivity in major depressive disorder. *Psychological Medicine*, *44*(10), 2053–2065. https://doi.org/10.1017/S0033291713002742

van Waarde, J. A., Scholte, H. S., van Oudheusden, L. J. B., Verwey, B., Denys, D., & van Wingen, G. A. (2015). A functional MRI marker may predict the outcome of electroconvulsive therapy in severe and treatment-resistant depression. *Molecular Psychiatry*, *20*(5), 609–614. https://doi.org/10.1038/mp.2014.78

Vasavada, M. M., Leaver, A. M., Espinoza, R. T., Joshi, S. H., Njau, S. N., Woods, R. P., & Narr, K. L. (2016). Structural connectivity and response to ketamine therapy in major depression: A preliminary study. *Journal of Affective Disorders*, *190*, 836–841. https://doi.org/10.1016/j.jad.2015.11.018

Vasavada, M. M., Loureiro, J., Kubicki, A., Sahib, A., Wade, B., Hellemann, G., … Leaver, A. M. (2020). Effects of Serial Ketamine Infusions on Corticolimbic Functional Connectivity in Major Depression. *Biological Psychiatry: Cognitive Neuroscience and Neuroimaging*, *6*(7), 735–744. https://doi.org/10.1016/j.bpsc.2020.06.015

Vidal, S., Jermann, F., Aubry, J.-M., Richard-Lepouriel, H., & Kosel, M. (2020). Effect of Ketamine on Rumination in Treatment-Resistant Depressive Patients. *Journal of Clinical Psychopharmacology*, *40*(6), 607–610. https://doi.org/10.1097/JCP.0000000000001305

Wade, B.S.C., Sui, J., Njau, S., Leaver, A. M., Vasvada, M., Gutman, B. A., … Joshi, S. H. (2017). Data-driven cluster selection for subcortical shape and cortical thickness predicts recovery from depressive symptoms. *Proceedings - International Symposium on Biomedical Imaging*. https://doi.org/10.1109/ISBI.2017.7950570

Wade, Benjamin S C, Hellemann, G., Espinoza, R. T., Woods, R. P., Joshi, S. H., Redlich, R., … Narr, K. L. (2020). Depressive Symptom Dimensions in Treatment-Resistant Major Depression and Their Modulation With Electroconvulsive Therapy. *The Journal of ECT*, *36*(2). Retrieved from https://journals.lww.com/ectjournal/Fulltext/2020/06000/Depressive_Symptom_Dimensions_in.10.aspx

Wade, Benjamin S C, Hellemann, G., Espinoza, R. T., Woods, R. P., Joshi, S. H., Redlich, R., … Narr, K. L. (2021). Accounting for symptom heterogeneity can improve neuroimaging models of antidepressant response after electroconvulsive therapy. *Human Brain Mapping*, *n/a*(n/a). https://doi.org/https://doi.org/10.1002/hbm.25620

Wang, L., Leonards, C. O., Sterzer, P., & Ebinger, M. (2014). White matter lesions and depression: a systematic review and meta-analysis. *Journal of Psychiatric Research*, *56*, 56–64. https://doi.org/10.1016/j.jpsychires.2014.05.005

Zacharias, N., Musso, F., Müller, F., Lammers, F., Saleh, A., London, M., … Winterer, G. (2020). Ketamine effects on default mode network activity and vigilance: A randomized, placebo-controlled crossover simultaneous fMRI/EEG study. *Human Brain Mapping*, *41*(1), 107–119. https://doi.org/10.1002/hbm.24791

Zarate Jr, C. A., Singh, J. B., Carlson, P. J., Brutsche, N. E., Ameli, R., Luckenbaugh, D. A., … Manji, H. K. (2006). A Randomized Trial of an N-methyl-D-aspartate Antagonist in Treatment-Resistant Major Depression. *Archives of General Psychiatry*, *63*(8), 856–864. https://doi.org/10.1001/archpsyc.63.8.856

Zhu, X., Wang, X., Xiao, J., Liao, J., Zhong, M., Wang, W., & Yao, S. (2012). Evidence of a dissociation pattern in resting-state default mode network connectivity in first-episode, treatment-naive major depression patients. *Biological Psychiatry*, *71*(7), 611–617. https://doi.org/10.1016/j.biopsych.2011.10.035

Figure/Table Legends

Figure 1. Boxplots of cross validated model performance. (a) Shows distributions of the sums-of-squares formulation of the coefficient of determination (R^2) in test data across 100 iterations of cross validation while (b) shows the normalized root mean squared error of predictions across cross validations.

Figure 2. Partial dependence plots showing the expected change in symptoms (y-axis) for observed values of informative imaging predictors (x-axis) while averaging other predictors. Associations between pretreatment values of the most informative predictors and expected symptom changes are shown for (a) HDRS-6 (top row) and Core Mood and Anhedonia (middle row) symptoms. The bottom row illustrates the location of the right posterior insula area 2 (PoI2), right anterior cingulate/medial prefrontal cortex (BA 10r), and right superior longitudinal fasciculus (SLF). Figure (b) illustrates the same for the rumination response scale: reflection associations with the posterior cingulate (v23ab subdivision), superior parietal cortex (7PL subdivision), and the granular insular cortex.

Figure S1. Scatter plots showing the predicted versus actual change along each set of symptom scales and subscales.

Table 1. Outline of patient demographic and clinical features.

Table 2. Subscales of the 17-item Hamilton Depression Rating Scale used in this study. Items marked with an "X" indicate the presence of the item in the respective subscale.

Table 3. Outline of the performance of each model measured by the R^2-score, error rate (NRMSE), and overall model significance adjusted for multiple comparisons.

Figure 1

Click here to access/download;Figure(s);Fig1.pdf

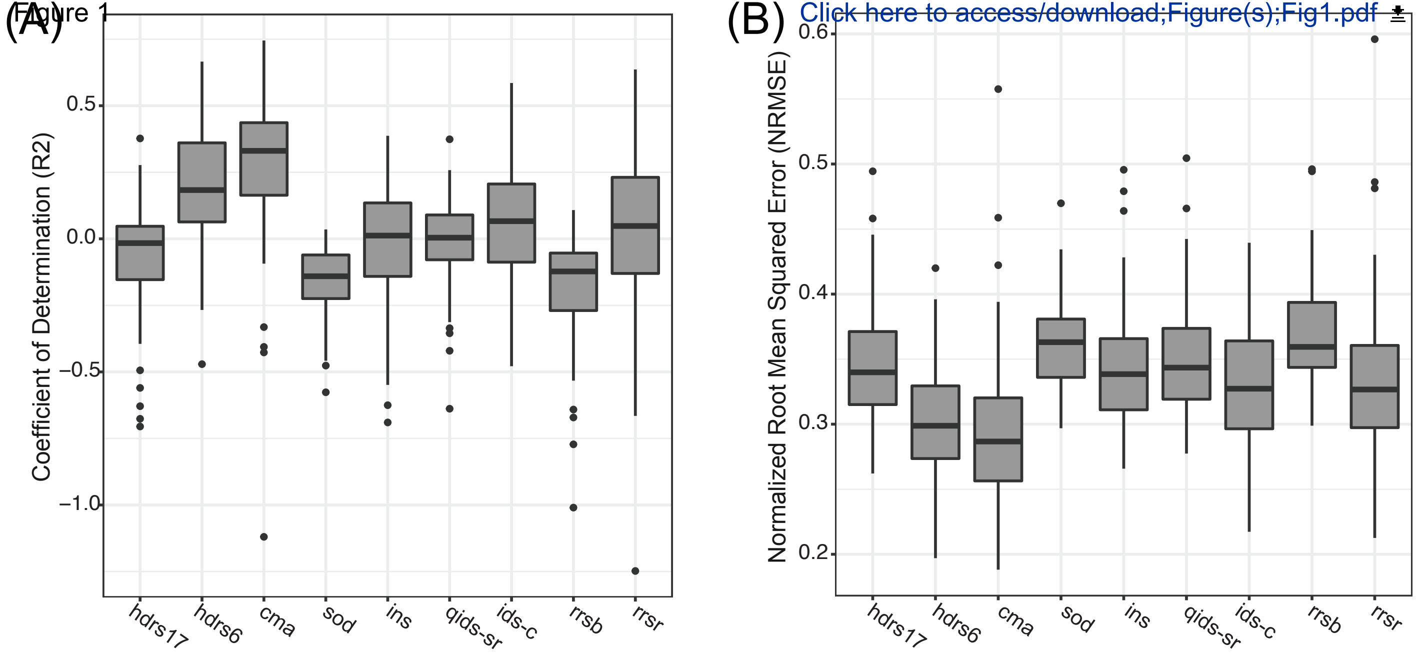

Click here to access/download;Table(s);Table_1_Demographic_Clinical_Data.

Table 1. Demographic and clinical outline

	Patients	Controls	p (T or χ^2)
N	60	19	
Age, mean (SD)	40.1 (11.1)	28.2 (6.8)	<0.0001
Sex, male/female	30/30	8/11	0.73
Lifetime illness duration, years, mean (SD)	24.8 (15.9)	N/A	--
Current illness duration, years, mean (SD)*	4.0 (5.9)	N/A	--
Number of lifetime depressive episodes, mean (SD)†	6.8 (14.4)	N/A	--
Bipolar disorder, yes/no	3/57	N/A	--
Clinical Rating Scales			
HDRS-17 baseline, mean (SD)	18.9 (4.6)	2.6 (5.1)	<0.0001
HDRS-17 change, mean (SD)	-10.6 (6.3)	-0.2 (2.1)	<0.0001
QIDS-SR baseline, mean (SD)	16.1 (4.8)	4.2 (6.2)	<0.0001
QIDS-SR change, mean (SD)	-9.5 (5.1)	-0.5 (1.3)	<0.0001
IDS-C baseline, mean (SD)	30.5 (7.4)	5.1 (9.4)	<0.0001
IDS-C change, mean (SD)	-17.6 (10.7)	-0.5 (2.7)	<0.0001
HDRS-17 Responder/Non-Responder, N	37/23	N/A	--
HDRS-17 Remitter/Non-Remitter, N	30/30	N/A	--
Medication History			
Current SSRI use, yes/no	18/42	N/A	--
Current SNRI use, yes/no	18/42	N/A	--
Current MAOI use, yes/no	2/58	N/A	--
Current Lithium use, yes/no	1/59	N/A	--
Current benzodiazepine use, yes/no	16/44	N/A	--
Current antipsychotic use, yes/no	13/47	N/A	--
Current use of any medication, yes/no	48/12	N/A	--
Education Level, N‡			
High School or Equivalent	3	0	--
Some College	23	3	--
Bachelor's	23	9	--
Masters'	14	3	--
Professional or Doctoral	6	3	--

* Three participants reported uncertain illness durations and were excluded from this calculation

† Thirty-five participants reported more episodes than they could count and were excluded from this calculation

‡ One patient and one control had incomplete data on education level

Click here to access/download;Table(s);Table_2_HDRS_Subscales.docx

Table 2. Subscales of the Hamilton Depression Rating Scale

HDRS-17 Items	HDRS-6	Core Mood & Anhedonia	Somatic Disturbances	Insomnia
Depressed Mood	X	X		
Feelings of Guilt	X		X	
Suicide				
Insomnia, Early				X
Insomnia, Middle				X
Insomnia, Late				X
Work and Activities	X	X		
Psychomotor Retardation	X	X		
Agitation			X	
Anxiety, Psychic	X		X	
Anxiety, Somatic			X	
Somatic Symptoms, GI			X	
Somatic Symptoms, General	X		X	
Genital Symptoms			X	
Hypochondriasis			X	
Weight Loss		X		
Insight				

Table 3
Click here to access/download;Table(s);Table_3_Model_Performance_Detaile

Table 3. Model Performance

**Outcome**	R^2	**NRMSE**	**Adjusted p-value (R^2)**
HDRS-17	-0.06	0.34	0.20
HDRS-6	0.19	0.30	0.02
CMA	0.27	0.29	0.02
SOD	-0.15	0.36	0.72
INS	-0.01	0.34	0.29
QIDS-SR	-0.01	0.35	0.07
IDS-C-M	0.04	0.33	0.06
RRSB	-0.17	0.36	0.72
RRSR	0.01	0.33	0.02

Table S1

Click here to access/download;Other Supplementary Material;Table_S1.xlsx

Thickness and Volume ROIs	Diffusion ROIs	Global Conne
lh_bankssts	Anterior_corona_radiata_L	L_V1
lh_caudalanteriorcingulate	Anterior_corona_radiata_R	L_MST
lh_caudalmiddlefrontal	Anterior_limb_IC_L	L_V6
lh_cuneus	Anterior_limb_IC_R	L_V2
lh_entorhinal	Body_corpus_callosum	L_V3
lh_fusiform	Cerebral_peduncle_L	L_V4
lh_inferiorparietal	Cerebral_peduncle_R	L_V8
lh_inferiortemporal	Cingulum_gyrus_L	L_4
lh_isthmuscingulate	Cingulum_gyrus_R	L_3b
lh_lateraloccipital	Cingulum_hippocampus_L	L_FEF
lh_lateralorbitofrontal	Cingulum_hippocampus_R	L_PEF
lh_lingual	Corticospinal_L	L_55b
lh_medialorbitofrontal	Corticospinal_R	L_V3A
lh_middletemporal	External_capsule_L	L_RSC
lh_parahippocampal	External_capsule_R	L_POS2
lh_paracentral	Fornix_Stria_terminalis_L	L_V7
lh_parsopercularis	Fornix_Stria_terminalis_R	L_IPS1
lh_parsorbitalis	Fornix_columnBody	L_FFC
lh_parstriangularis	Genu_corpus_callosum	L_V3B
lh_pericalcarine	Inferior_cerebellar_peduncle_L	L_LO1
lh_postcentral	Inferior_cerebellar_peduncle_R	L_LO2
lh_posteriorcingulate	Medial_lemniscus_L	L_PIT
lh_precentral	Medial_lemniscus_R	L_MT
lh_precuneus	Middle_cerebellar_peduncle	L_A1
lh_rostralanteriorcingulate	Pontine_crossing_tract_partOfMCP	L_PSL
lh_rostralmiddlefrontal	Posterior_corona_radiata_L	L_SFL
lh_superiorfrontal	Posterior_corona_radiata_R	L_PCV
lh_superiorparietal	Posterior_limb_IC_L	L_STV
lh_superiortemporal	Posterior_limb_IC_R	L_7Pm
lh_supramarginal	Posterior_thalamic_radiation_L	L_7m
lh_frontalpole	Posterior_thalamic_radiation_R	L_POS1
lh_temporalpole	Retrolenticular_IC_L	L_23d
lh_transversetemporal	Retrolenticular_IC_R	L_v23ab
lh_insula	SLF_L	L_d23ab
rh_bankssts	SLF_R	L_31pv
rh_caudalanteriorcingulate	Sagittal_stratum_ILF_IFOF_L	L_5m
rh_caudalmiddlefrontal	Sagittal_stratum_ILF_IFOF_R	L_5mv
rh_cuneus	Splenium_corpus_callosum	L_23c
rh_entorhinal	Superior_cerebellar_peduncle_L	L_5L
rh_fusiform	Superior_cerebellar_peduncle_R	L_24dd
rh_inferiorparietal	Superior_corona_radiata_L	L_24dv
rh_inferiortemporal	Superior_corona_radiata_R	L_7AL

rh_isthmuscingulate	Superior_fronto_occipital_fasciculus_L	L_SCEF
rh_lateraloccipital	Superior_fronto_occipital_fasciculus_R	L_6ma
rh_lateralorbitofrontal	Tapetum_L	L_7Am
rh_lingual	Tapetum_R	L_7PL
rh_medialorbitofrontal	Uncinate_fasciculus_L	L_7PC
rh_middletemporal	Uncinate_fasciculus_R	L_LIPv
rh_parahippocampal		L_VIP
rh_paracentral		L_MIP
rh_parsopercularis		L_1
rh_parsorbitalis		L_2
rh_parstriangularis		L_3a
rh_pericalcarine		L_6d
rh_postcentral		L_6mp
rh_posteriorcingulate		L_6v
rh_precentral		L_p24pr
rh_precuneus		L_33pr
rh_rostralanteriorcingulate		L_a24pr
rh_rostralmiddlefrontal		L_p32pr
rh_superiorfrontal		L_a24
rh_superiorparietal		L_d32
rh_superiortemporal		L_8BM
rh_supramarginal		L_p32
rh_frontalpole		L_10r
rh_temporalpole		L_47m
rh_transversetemporal		L_8Av
rh_insula		L_8Ad
Left_Lateral_Ventricle		L_9m
Left_Cerebellum_White_Matter		L_8BL
Left_Thalamus_Proper		L_9p
Left_Caudate		L_10d
Left_Putamen		L_8C
Left_Pallidum		L_44
Brain_Stem		L_45
Left_Hippocampus		L_47l
Left_Amygdala		L_a47r
Left_Accumbens_area		L_6r
Right_Lateral_Ventricle		L_IFJa
Right_Cerebellum_White_Matter		L_IFJp
Right_Thalamus_Proper		L_IFSp
Right_Caudate		L_IFSa
Right_Putamen		L_p9_46v
Right_Pallidum		L_46
Right_Hippocampus		L_a9_46v

Right_Amygdala	L_9_46d
Right_Accumbens_area	L_9a
CC_Posterior	L_10v
CC_Mid_Posterior	L_a10p
CC_Central	L_10pp
CC_Mid_Anterior	L_11l
CC_Anterior	L_13l
	L_OFC
	L_47s
	L_LIPd
	L_6a
	L_i6_8
	L_s6_8
	L_43
	L_OP4
	L_OP1
	L_OP2_3
	L_52
	L_RI
	L_PFcm
	L_PoI2
	L_TA2
	L_FOP4
	L_MI
	L_Pir
	L_AVI
	L_AAIC
	L_FOP1
	L_FOP3
	L_FOP2
	L_PFt
	L_AIP
	L_EC
	L_PreS
	L_H
	L_ProS
	L_PeEc
	L_STGa
	L_PBelt
	L_A5
	L_PHA1
	L_PHA3
	L_STSda

L_STSdp
L_STSvp
L_TGd
L_TE1a
L_TE1p
L_TE2a
L_TF
L_TE2p
L_PHT
L_PH
L_TPOJ1
L_TPOJ2
L_TPOJ3
L_DVT
L_PGp
L_IP2
L_IP1
L_IP0
L_PFop
L_PF
L_PFm
L_PGi
L_PGs
L_V6A
L_VMV1
L_VMV3
L_PHA2
L_V4t
L_FST
L_V3CD
L_LO3
L_VMV2
L_31pd
L_31a
L_VVC
L_25
L_s32
L_pOFC
L_PoI1
L_Ig
L_FOP5
L_p10p
L_p47r

L_TGv
L_MBelt
L_LBelt
L_A4
L_STSva
L_TE1m
L_PI
L_a32pr
L_p24
R_V1
R_MST
R_V6
R_V2
R_V3
R_V4
R_V8
R_4
R_3b
R_FEF
R_PEF
R_55b
R_V3A
R_RSC
R_POS2
R_V7
R_IPS1
R_FFC
R_V3B
R_LO1
R_LO2
R_PIT
R_MT
R_A1
R_PSL
R_SFL
R_PCV
R_STV
R_7Pm
R_7m
R_POS1
R_23d
R_v23ab
R_d23ab

R_31pv
R_5m
R_5mv
R_23c
R_5L
R_24dd
R_24dv
R_7AL
R_SCEF
R_6ma
R_7Am
R_7PL
R_7PC
R_LIPv
R_VIP
R_MIP
R_1
R_2
R_3a
R_6d
R_6mp
R_6v
R_p24pr
R_33pr
R_a24pr
R_p32pr
R_a24
R_d32
R_8BM
R_p32
R_10r
R_47m
R_8Av
R_8Ad
R_9m
R_8BL
R_9p
R_10d
R_8C
R_44
R_45
R_47l
R_a47r

R_6r
R_IFJa
R_IFJp
R_IFSp
R_IFSa
R_p9_46v
R_46
R_a9_46v
R_9_46d
R_9a
R_10v
R_a10p
R_10pp
R_11l
R_13l
R_OFC
R_47s
R_LIPd
R_6a
R_i6_8
R_s6_8
R_43
R_OP4
R_OP1
R_OP2_3
R_52
R_RI
R_PFcm
R_PoI2
R_TA2
R_FOP4
R_MI
R_Pir
R_AVI
R_AAIC
R_FOP1
R_FOP3
R_FOP2
R_PFt
R_AIP
R_EC
R_PreS
R_H

R_ProS
R_PeEc
R_STGa
R_PBelt
R_A5
R_PHA1
R_PHA3
R_STSda
R_STSdp
R_STSvp
R_TGd
R_TE1a
R_TE1p
R_TE2a
R_TF
R_TE2p
R_PHT
R_PH
R_TPOJ1
R_TPOJ2
R_TPOJ3
R_DVT
R_PGp
R_IP2
R_IP1
R_IP0
R_PFop
R_PF
R_PFm
R_PGi
R_PGs
R_V6A
R_VMV1
R_VMV3
R_PHA2
R_V4t
R_FST
R_V3CD
R_LO3
R_VMV2
R_31pd
R_31a
R_VVC

R_25
R_s32
R_pOFC
R_PoI1
R_Ig
R_FOP5
R_p10p
R_p47r
R_TGv
R_MBelt
R_LBelt
R_A4
R_STSva
R_TE1m
R_PI
R_a32pr
R_p24
accumbens_le
accumbens_r
amygdala_lef
amygdala_rig
caudate_left
caudate_right
cerebellum_le
cerebellum_r
diencephalon
diencephalon
hippocampus
hippocampus
pallidum_left
pallidum_righ
putamen_left
putamen_righ
thalamus_left
thalamus_righ
brainStem
Habennula_L
Habennula_R

ctivity ROIs

eft
ight
t
ht

t
eft
ight
_left
_right
_left
_right

nt
[illegible]
nt
t
ht

CONSORT Diagram
Click here to access/download;Other Supplementary Material;CONSORT_Diagram.doc

CONSORT
TRANSPARENT REPORTING of TRIALS

CONSORT 2010 Flow Diagram

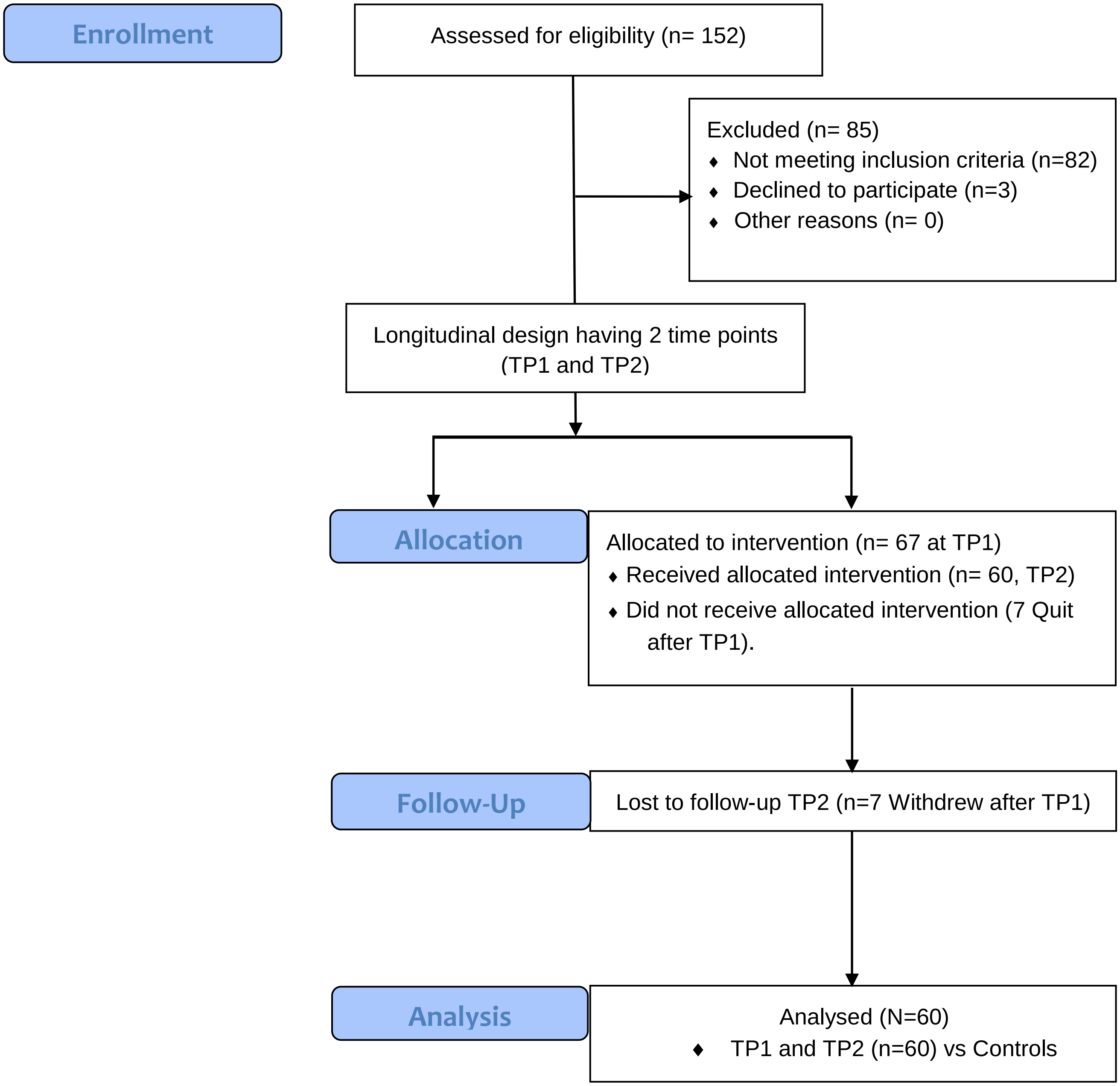

Click here to access/download;Other Supplementary Material;CONSORT 2010 Checklist.doc

CONSORT 2010 checklist of information to include when reporting a randomised trial*

Section/Topic	Item No	Checklist item	Reported on page No
**Title and abstract**			
	1a	Identification as a randomised trial in the title	NA
	1b	Structured summary of trial design, methods, results, and conclusions (for specific guidance see CONSORT for abstracts)	2
**Introduction**			
Background and objectives	2a	Scientific background and explanation of rationale	3, 4 & 5
	2b	Specific objectives or hypotheses	5
**Methods**			
Trial design	3a	Description of trial design (such as parallel, factorial) including allocation ratio	5 & 6
	3b	Important changes to methods after trial commencement (such as eligibility criteria), with reasons	NA
Participants	4a	Eligibility criteria for participants	6 & 7
	4b	Settings and locations where the data were collected	7
Interventions	5	The interventions for each group with sufficient details to allow replication, including how and when they were actually administered	7
Outcomes	6a	Completely defined pre-specified primary and secondary outcome measures, including how and when they were assessed	9
	6b	Any changes to trial outcomes after the trial commenced, with reasons	NA
Sample size	7a	How sample size was determined	5 https://clinicaltrials.gov/ct2/show/NCT02165449
	7b	When applicable, explanation of any interim analyses and stopping guidelines	NA
Randomisation:			
Sequence generation	8a	Method used to generate the random allocation sequence	NA
	8b	Type of randomisation; details of any restriction (such as blocking and block size)	NA
Allocation concealment	9	Mechanism used to implement the random allocation sequence (such as sequentially numbered containers), describing any steps taken to conceal the sequence until interventions were assigned	NA

mechanism			
Implementation	10	Who generated the random allocation sequence, who enrolled participants, and who assigned participants to interventions	NA
Blinding	11a	If done, who was blinded after assignment to interventions (for example, participants, care providers, those assessing outcomes) and how	NA
	11b	If relevant, description of the similarity of interventions	NA
Statistical methods	12a	Statistical methods used to compare groups for primary and secondary outcomes	9
	12b	Methods for additional analyses, such as subgroup analyses and adjusted analyses	NA
**Results**			
Participant flow (a diagram is strongly recommended)	13a	For each group, the numbers of participants who were randomly assigned, received intended treatment, and were analysed for the primary outcome	NA
	13b	For each group, losses and exclusions after randomisation, together with reasons	NA
Recruitment	14a	Dates defining the periods of recruitment and follow-up	6
	14b	Why the trial ended or was stopped	NA
Baseline data	15	A table showing baseline demographic and clinical characteristics for each group	6
Numbers analysed	16	For each group, number of participants (denominator) included in each analysis and whether the analysis was by original assigned groups	8
Outcomes and estimation	17a	For each primary and secondary outcome, results for each group, and the estimated effect size and its precision (such as 95% confidence interval)	10
	17b	For binary outcomes, presentation of both absolute and relative effect sizes is recommended	NA
Ancillary analyses	18	Results of any other analyses performed, including subgroup analyses and adjusted analyses, distinguishing pre-specified from exploratory	10 & 11
Harms	19	All important harms or unintended effects in each group (for specific guidance see CONSORT for harms)	NA
**Discussion**			
Limitations	20	Trial limitations, addressing sources of potential bias, imprecision, and, if relevant, multiplicity of analyses	16
Generalisability	21	Generalisability (external validity, applicability) of the trial findings	16
Interpretation	22	Interpretation consistent with results, balancing benefits and harms, and considering other relevant evidence	12, 13, 14, 15 & 16
**Other information**			
Registration	23	Registration number and name of trial registry	2
Protocol	24	Where the full trial protocol can be accessed, if available	2
Funding	25	Sources of funding and other support (such as supply of drugs), role of funders	16

*We strongly recommend reading this statement in conjunction with the CONSORT 2010 Explanation and Elaboration for important clarifications on all the items. If relevant, we also recommend reading CONSORT extensions for cluster randomised trials, non-inferiority and equivalence trials, non-pharmacological treatments, herbal interventions, and pragmatic trials. Additional extensions are forthcoming: for those and for up to date references relevant to this checklist, see www.consort-statement.org.

Reviewers' and editor's comments:

<u>Reviewer #1:</u> Wade et al. used purely data-driven machine learning models to predict individual reductions across multiple depression scales following treatment, using pretreatment multimodal RSFC, structural neuroimaging and demographic data. They state that ketamine infusion generally preserves or enhances cortico-subcortical connectivity patterns, disrupting cortico-cortical connectivity and normalizing aberrant somato-motor and default mode network (DMN) connectivity. Wade et al. compared a group of 60 MD patients, receiving a series of four ketamine infusions, to untreated and unaffected controls. The most informative features in the prediction of overall symptom changes following serial ketamine injection were the pretreatment global connectivity of the right posterior insula, subdivisions of the anterior cingulate cortex (ACC), the medial prefrontal cortex (mPFC), and the diffusion kurtosis of the right superior longitudinal fasciculus (SLF). Wade and colleagues conclude their study, by stressing the high robustness of positive effects observed after application of ketamine, however highlighting relevant avenues of improvement.

This review offers some clear overall strengths:

An innovative and state-of-the-art statistical approach using predictive multivariate machine learning procedures, rather than relying on univariate correlations
A coherent and well-balanced depiction of predicted vs. actual changes in relevant MD symptom dimensions
Concise and exhaustive discussion of observed effects across all clusters of depressive symptoms
A special focus was taken on a wide array of neuroimaging measures (structural, resting state functional & DTI) and neurological structures as potential biomarkers for individual differences in susceptibility to ketamine treatment
Although not RCT, the use of CONSORT trial-reporting standards is very commendable and adds important transparency

In total, Wade et al present clear and meaningful interpretations of their findings, not refraining from pointing out inherent weaknesses, and providing elaborate solutions for future research.

There is only one minor hint:

Authors might consider including the review by Alario & Niciu (2021) "Biomarkers of ketamine's antidepressant effect: a clinical review of genetics, functional connectivity, and neurophysiology" (10.1177/24705470211014210) into their discussion or introduction.

Response: We thank the reviewer for their kind review and insightful comments. We have now referenced the excellent review by Alario and Niciu in the introduction stating, "A recent review by Alario & Niciu surveyed genetic, RSFC, neurophysiological predictors of response to ketamine. The authors reported that ketamine generally normalized disrupted functional connectivity in patients with MDD, though study-specific results have varied and have largely failed to be replicated. Despite this, connectivity of the insula, anterior cingulate, and left amygdala were widely reported in relation to response to ketamine (Alario & Niciu, 2021)."

Wade et al's study is important groundwork for the understanding of how biological predispositions should inform individual treatment decisions. The authors rightly stress the importance of identifying treatment-response biomarkers of antidepressant outcomes, especially for rapidly-acting antidepressant treatments. I am convinced, that the identification of such biomarkers may be of substantial benefit for advancing the

development of personalized treatment strategies for ketamine and potentially also for other fast-acting therapies. I can therefore recommend this paper for publication in its current form.

Response: We are very grateful for this positive feedback.

Reviewer #2: This study is to explore the multimodal MRI indicators including cortical thickness, subcortical volume, diffusion measurement, and functional global connectivity before receiving Serial Ketamine Infusion treatment for depression patients to predict the improvement of symptoms of depression patients by Ketamine treatment. The results of random forest regression models revealed that increased right mPFC/ACC and PoI and lower kurtosis of the superior longitudinal fasciculus predicted reduced HDRS-6 and CMA symptoms following treatment. This research is innovative and has certain application significance. However, there are some issues that need to be addressed more specifically before this manuscript can be published.

Comment 1: In the introduction, the author made hypotheses about key limbic structures such as the hippocampus in the clinical outcomes following SKI, but the results of the study did not verify this hypothesis. The author should explain this inconsistency in the discussion section.

Response: We thank the reviewer for pointing out this oversight. We have added a paragraph at the end of the discussion section to address this. We state:

"Lastly, it was hypothesized that hippocampal volume or connectivity would be a predictor of response to SKI given that it has been associated with response to other treatments including electroconvulsive therapy (B.S.C. Wade et al., 2017) and pharmaceutical treatments (Colle et al., 2018). Further, the effects of depression on hippocampal structure have been widely reported (Sheline, Wang, Gado, Csernansky, & Vannier, 1996) making its structural or functional properties a plausible candidate biomarker of treatment response. Contrary to our expectations, however, hippocampal structure and function was not implicated as a predictor of response to SKI in this multivariate framework. It is possible that this may reflect distinct mechanisms of actions for SKI or a predominating association of more salient predictors in a multivariate framework that overshadow the contributions of the hippocampus."

Comment 2: The lack of information in the control group confuses readers, including the inclusion criteria and whether to accept ketamine infusion, etc. The author should list it.

Response: We apologize if the information provided regarding the control group was ambiguous. To clarify, the non-depressed controls were studied without any intervention. Depressed participants determined as clinically eligible to receive ketamine therapy, were given the opportunity to seek other forms of depression treatment or no-treatment rather than participate in this clinical trial during the informed consent process. In the revised manuscript, we now outline the full inclusion criteria for non-depressed control participants stating, "Inclusion criteria for non-depressed control participants were an age between 20-64 years, no history of depressive disorder or bipolar disorder that is current, recurrent, or with a single episode that lasted longer than one year, no use antidepressants or mood stabilizers within the past 6 months, ability to read and understand English, and an ability to provide informed consent." Exclusion criteria included current or past depression diagnoses or any other Axis 1 disorder, and other criteria that overlapped with the patient participants (e.g., substance abuse, mental retardation, dementia, neurological condition or other major medical illness, pregnancy, contraindication to scanning).

Comment 3: The author should list the statistical analysis methods between the groups, mapping software, and corresponding literature support, which makes the research more reliable

Response: This point is well taken. We have updated the "Predictive Features" section of the paper to describe the mapping/processing software used here:

"Demographic (age and sex) and pretreatment multimodal imaging features were included as predictors. Multimodal imaging data were visually inspected and preprocessed using HCP minimal pipelines (Glasser et al., 2013; Stephen M Smith et al., 2013) with the BIDS-App (Gorgolewski et al., 2017). Functional imaging artifacts were removed using a modified spatial independent components analysis (sICA) method (Griffanti et al., 2014) and FSL's FIX (https://fsl.fmrib.ox.ac.uk/fsl/fslwiki/FIX). MSMall registration aligned cortical regions using measures of cortical folding, thickness, myelination, and resting-state connectivity information (Glasser et al., 2016; Robinson et al., 2018, 2014). Resting-state time series data was represented on the cortical surface and the time series was averaged across AP and PA acquisitions. Structural imaging features included regional estimates of cortical thickness in 68 regions based on the Desikan-Killiany FreeSurfer atlas (Desikan et al., 2006), 24 subcortical volumes, diffusion measures (fractional anisotropy (FA), radial (RD), axial (AD), mean diffusivity (MD), and diffusion kurtosis (DK)) of 48 white matter tracts. NiLearn scripts were used to compute functional global connectivity measures of 360 cortical and 21 subcortical regions. A tabulation of regional predictors is included in Table S1."

Between diagnostic group statistical analyses were not the focus of this paper, but rather included to determine the variance associated with repeat assessments and characterize normative values. Consequently, these contrasts were limited only to comparisons of symptom reductions between patients with depression and unaffected controls who received no intervention (see the results section: Cohort Characteristics). To compare symptom changes between groups, we simply used unpaired, two-sample t-tests. We now note this in the Cohort Characteristics section stating, *"We used unpaired, two-sample t-tests to compare the degree of symptom changes between patients and controls. As expected, symptoms captured by the HDRS-17, IDS-C, and QIDS-SR, were significantly more reduced among patients than controls (all $p<0.0001$)."*

Comment 4: In the Evaluation of Confounding Variables section, the author uses the t-tests and Pearson correlation tests method,

1. whether the author conducts a normality test of the data.
2. Whether gender, age, education level, course of disease and other factors are included in the Pearson correlation tests method as covariates to exclude their influence.
3. When the author calculated the predictive effect of multiple imaging indicators on the clinical outcome of treatment, whether confounding factors such as age, gender, education level, and disease course were excluded as covariates.

Response: We thank the reviewer for this inquiry. We address these comments point-by-point:

1. *"whether the author conducts a normality test of the data."* For the primary analysis, we note that random forest models are non-parametric and robust to skewed data; thus, no normality tests were conducted or needed for the predictive models. To address normality assumptions for linear models evaluating confounds, we note that global connectivity measures (e.g., PoI2 and 10r) are count measures; that is, they are integer values reflecting the number of regions that these regions are functionally correlated with above the threshold $r \geq |0.3|$. In recognition of this, we have adjusted the approach to evaluate confounding variables to use Poisson regression for global connectivity outcomes. Standard linear models are used for other outcomes (e.g., SLF diffusivity). The kurtosis of the right SLF was normally distributed according to a Shapiro-Wilk test ($p>0.05$).
2. *"Whether gender, age, education level, course of disease and other factors are included in the Pearson correlation tests method as covariates to exclude their influence"*. To determine whether these variables are significantly associated with the brain regional measures that were predictive in the random forest models, we used Poisson or ordinary least squares regression models. Here, each region's property (connectivity, diffusivity, or thickness) was the dependent variable with a single

predictor included. Related to this, we have now updated our demographic table and exploration of confounds to include education level.

3. *"When the author calculated the predictive effect of multiple imaging indicators on the clinical outcome of treatment, whether confounding factors such as age, gender, education level, and disease course were excluded as covariates."*

We have updated our evaluation of confounding variables to include evaluation of updated models that include all demographic and clinical variables, in addition to multimodal imaging predictors. Here, we found that inclusion of new predictors (current medication use [including SSRI, SNRI, antipsychotics, and benzodiazapines], education level, and depressive episode duration) did not improve our model performance. We also observed that none of these predictors were among the top informative features in models that included imaging measures. Thus, we conclude that they did not improve our prediction of clinical outcomes despite some associations with predictive imaging measures. We have updated the Evaluation of Confounding Variables section as follows:

"We evaluated whether predictive imaging measures outlined above (PoI2, BA 10r, SLF DK, BA 7PL, left insular gyrus, and left v23ab) and significantly-predicted outcomes were associated with potential confounding clinical and demographic variables. Measures of global connectivity are based on counts, thus we evaluated associations with potential confounding variables using Poisson regression. The diffusivity of the SLF and thickness of the left insular gyrus were normally distributed (following a Shapiro-Wilk test; $p>0.05$) and continuous measures; thus, associations with confounds were evaluated using standard linear regression models. Global connectivity of the right PoI2 was associated with age, sex, education, duration of current episode, and current SSRI, SNRI, and benzodiazepine use (all $p<0.05$). Global connectivity of right BA 10r was associated with age, sex, duration of current episode, and current SNRI use (all $p<0.05$). Global connectivity of right BA 7PL was associated with sex, education, current episode duration, and current SSRI use (all $p<0.05$). Left area v23ab was associated with current episode duration, and current use of antipsychotics and SNRIs (all $p<0.05$). The kurtosis of the right SLF was not associated with the evaluated confounding variables. The left insula was associated with patient age and SSRI use (all $p<0.05$). We reran the original RFR models with all of the original predictors with the addition of medication use history, depressive episode duration, and education level. T-tests revealed no significant differences between original and updated model performance upon inclusion of additional confounding variables (all $p>0.9$). Further, no potential confounding clinical or demographic variables were among important predictors in the updated models. Thus, despite their associations with important imaging predictors, these potentially confounding variables did not improve prediction of outcomes for any dimension."

Comment 5: In this study, 48/60 people have a history of using drugs. How did the author identify and exclude the effects of the current drugs on the observation indicators.

Response: Notably, eligibility criteria for all participants in this study included a negative urine drug screen for illicit drug use, a normal comprehensive metabolic panel of kidney and liver function, blood pressure measurement and 12 lead ECG of heart function within a week of initiating treatment. However, for patients, the use of stable (> 6 weeks) antidepressant medication was allowed. As was the case for other potentially confounding variables explored in the "Evaluation of Confounding Variables" section, we conducted t-tests to determine whether symptom changes were associated with current medication use. We note in this section that "Change in the HDRS-6, CMA, or RRSR symptoms was not associated with the above [*including current medications*] confounding variables apart from an association between the HDRS-6 change and age." We note in the *Participants* section the following, "All participants received measurement of vital signs (e.g., BP, HR, temperature), a blood draw to determine metabolic, kidney and liver function, EKG, and provided a urine sample for drug and pregnancy (women only) screening. Drug

and pregnancy screens were required to be negative, and all lab results were reviewed by the study physician to ensure there were no contraindications to participating in the study."

Comment 6: The layout of the flowchart and the inconsistent lines in the supplementary material, the author needs to work hard to make it more perfect.

Response: We thank the reviewer for this observation and have adjusted the CONSORT Diagram accordingly.

DEPARTMENT OF NEUROLOGY
DAVID GEFFEN SCHOOL OF MEDICINE AT UCLA
10833 LE CONTE AVE, ROOM 16-131
LOS ANGELES, CA 90095

Editor-in-Chief
Kenneth S. Kendler, MD

Dec 20, 2021

Dear Dr. Kendler,

We would like to express our sincere appreciation for the insightful reviews we received for our manuscript **"Anterior Default Mode Network and Posterior Insular Connectivity is Predictive of Depressive Symptom Reduction Following Serial Ketamine Infusion"** (manuscript number: **PSM-D-21-01672**). We are thankful for the chance to clarify several issues that were noted by the reviewers and we believe that the changes made have greatly improved the content and clarity of the manuscript.

We have addressed the reviewers' comments on a point-by-point basis and have revised our manuscript as stated in the responses. Each of the reviewers' critiques is followed by our response and the specific changes made to the manuscript.

Please address all correspondences concerning this work to me by email at Benjamin.SC.Wade@gmail.com.

Sincerely,

Ben Wade

Benjamin

Benjamin Wade, PhD
Postdoctoral Fellow, Department of Neurology
Geffen School of Medicine at UCLA
