# Supplementary material for: Anterior default mode network and posterior insular connectivity is predictive of depressive symptom reduction following serial ketamine infusion
Source: Psychol Med. 2022 May 17;52(12):2376–86. doi: 10.1017/S0033291722001313 (PMC9527672; doi:10.1017/S0033291722001313)
Supplement: Supplementary file 1 [file S0033291722001313sup.zip › S0033291722001313sup002.doc]

**CONSORT 2010 Flow Diagram**

**Allocation**

**Analysis**

**Follow-Up**

**Enrollment**

Assessed for eligibility (n= 152)

Excluded (n= 85)

♦  Not meeting inclusion criteria (n=82)

♦  Declined to participate (n=3)

♦  Other reasons (n= 0)

Lost to follow-up TP2 (n=7 Withdrew after TP1)

Allocated to intervention (n= 67 at TP1)

♦ Received allocated intervention (n= 60, TP2)

♦ Did not receive allocated intervention (7 Quit after TP1).

Analysed (N=60)

- TP1 and TP2 (n=60) vs Controls (n=19)

Longitudinal design having 2 time points (TP1 and TP2)

(
